# PolyQ-expanded ataxin-2 aggregation impairs cellular processing-body homeostasis *via* sequestering the RNA helicase DDX6

**DOI:** 10.1016/j.jbc.2024.107413

**Published:** 2024-05-27

**Authors:** Jian-Yang Wang, Ya-Jun Liu, Xiang-Le Zhang, Yin-Hu Liu, Lei-Lei Jiang, Hong-Yu Hu

**Affiliations:** 1 State Key Laboratory of Molecular Biology, Shanghai Institute of Biochemistry and Cell Biology, Center for Excellence in Molecular Cell Science, Chinese Academy of Sciences, Shanghai, PR China; 2 University of Chinese Academy of Sciences, Beijing, PR China

**Keywords:** ataxin-2, aggregation, sequestration, P-bodies, DDX6, mRNA decay, translational repression

## Abstract

Ataxin-2 (Atx2) is a polyglutamine (polyQ) tract-containing RNA-binding protein, while its polyQ expansion may cause protein aggregation that is implicated in the pathogenesis of neurodegenerative diseases such as spinocerebellar ataxia type 2 (SCA2). However, the molecular mechanism underlying how Atx2 aggregation contributes to the proteinopathies remains elusive. Here, we investigated the influence of Atx2 aggregation on the assembly and functionality of cellular processing bodies (P-bodies) by using biochemical and fluorescence imaging approaches. We have revealed that polyQ-expanded (PQE) Atx2 sequesters the DEAD-box RNA helicase (DDX6), an essential component of P-bodies, into aggregates or puncta *via* some RNA sequences. The N-terminal like-Sm (LSm) domain of Atx2 (residues 82–184) and the C-terminal helicase domain of DDX6 are responsible for the interaction and specific sequestration. Moreover, sequestration of DDX6 may aggravate pre-mRNA mis-splicing, and interfere with the assembly of cellular P-bodies, releasing the endoribonuclease MARF1 that promotes mRNA decay and translational repression. Rescuing the DDX6 protein level can recover the assembly and functionality of P-bodies, preventing targeted mRNA from degradation. This study provides a line of evidence for sequestration of the P-body components and impairment of the P-body homeostasis in dysregulating RNA metabolism, which is implicated in the disease pathologies and a potential therapeutic target.

Protein aggregation is generally thought to be closely associated with the pathogenesis of neurodegenerative diseases (1), while polyglutamine (polyQ) expansion can drive amyloidogenic aggregation of the proteins, which is one of the typical causative factors that feature the disease pathologies (2, 3, 4). Human ataxin-2 (Atx2, also abbreviated to ATXN2) is such a polyQ tract-containing protein (5); expansion of the polyQ tract in Atx2 can trigger protein aggregation that may cause neurodegenerative diseases like spinocerebellar ataxia type 2 (SCA2) (6, 7, 8, 9).

Atx2 is a ubiquitous cytoplasmic protein that is highly conserved in eukaryotes from yeast to mammals (5, 10). Earlier studies suggested that human Atx2 contains 1313 amino-acid residues in full length (5, 10). However, a second functional start codon located at the fifth residue upstream of the *CAG* repeats is considered an updated version (11, 12), resulting in a shortened Atx2 protein with 1153 residues deposited in NCBI (NM_002973.4). Atx2 is a member of the like-Sm (LSm) protein family and is comprised of an N-terminal polyQ tract, an LSm domain, and an LSm-associated domain (LSmAD) (13, 14), which are involved in many aspects of cellular metabolism (12). In addition, Atx2 also contains a poly(A)-binding protein (PABP)-interacting motif 2 (PAM2) in the middle region, which mediates the interaction with the C-terminal mademoiselle (MLLE) domain of poly(A)-binding protein C1 (PABPC1) (15), suggesting that Atx2 might participate in the regulation of poly(A) tailing, mRNA decay and translation in cytoplasm (16). In addition, Atx2 is an RNA-binding protein (RBP) with diverse cellular functions, including assemblies of stress granules (SGs) and processing bodies (P-bodies) (17, 18, 19), RNA regulation (20), lipid metabolism (21), calcium homeostasis (22, 23), and endoplasmic reticulum (ER) dynamics and morphology (24, 25). The polyQ-tract length within normal Atx2 is 22 to 23 glutamines, but in SCA2 patients, much longer repeats (more than 34 repeats) are found in the Atx2 foci, and it may expand with successive generations (26, 27, 28). Intriguingly, the intermediate length of the polyQ tract (27–33 glutamines) has been reported to be associated with amyotrophic lateral sclerosis (ALS) (29, 30). Similar to other polyQ proteins, the expanded polyQ tract has been proposed to promote Atx2 cytotoxicity and cause various neurodegenerative disorders (3). However, the molecular mechanism underlying how polyQ-expanded (PQE) Atx2 leads to proteinopathies remains elusive.

Eukaryotic cells are composed of membrane-bound and membraneless organelles (31, 32, 33). These membraneless organelles include nucleoli, Cajal bodies, germ granules, SGs, and P-bodies (31). The lack of surrounding membrane entitles the organelles to be more dynamic and functionally tunable (34). It has been reported that Atx2 is associated with several membraneless organelles (17, 18). P-bodies are cytoplasmic ribonucleoprotein (RNP) granules containing various components, including mRNAs in complex with proteins and microRNAs engaged in mRNA decay and translational repression (35). They are evolutionarily conserved dynamic granules found in yeast that bear similarities to other RNP granules (33). P-bodies contain more than 42 factors including decapping enzymes and their associated activators, deadenylation factors (36), mRNA decay factors (37), microRNA (38), and translation regulators (39, 40). Composition analysis suggests that P-bodies play important roles in chromatin regulation, RNA processing, protein synthesis, and protein degradation (41), but the clear function of P-bodies in translational repression remains contentious.

Based on sequence homology that Atx2 and some other RBPs share an LSm domain or a PAM2 motif (5), Atx2 associates with RNA metabolism by forming a complex with other known RBPs (14), including Staufen1 (19), DEAD-box RNA helicase 6 (DDX6, also termed RCK/P54) (17), and TDP-43 (29). However, it is still poorly understood how these RBPs contribute to the biological function of the Atx2 complex. In addition, Atx2 is a constituent protein of SGs and P-bodies (17, 18, 42, 43), while downregulation of Atx2 may impair their formation (12, 44), suggesting its function in regulating RNA metabolism and protein translation. Moreover, poly(A)-binding protein-binding protein 1 (Pbp1), the yeast ortholog of Atx2, participates in the formation of SGs (14). Although SGs and P-bodies are considered in distinct organelles with different RBPs, a percentage of SGs and P-bodies are found to be docked from one another in a dynamic manner to share RBPs and mRNAs (44, 45), further demonstrating that these two membraneless organelles are structurally analogous and functionally linked.

To understand the impact of Atx2 aggregation on cellular homeostasis, we focused on elucidating the molecular mechanism underlying how Atx2 aggregation and sequestration lead to the deterioration in the structure and function of P-bodies. It was previously reported that overexpression of Atx2 interferes with the assembly of P-bodies *via* interacting with DDX6 (17). In this study, we showed that PQE Atx2 sequesters DDX6 into aggregates or puncta through the association of its N-terminal LSm domain with the C-terminal domain of DDX6, which is also mediated by the RNAs with particular sequences. As DDX6 is a key component of P-bodies (46, 47), sequestration of DDX6 by PQE Atx2 aggregates exacerbates MARF1 (meiosis regulator and mRNA stability factor 1)-mediated mRNA decay and consequently triggers translational repression through disrupting the P-body assembly. Thus, we proposed an alternative mechanism underlying how PQE Atx2 aggregation dysregulates cytoplasmic RNA metabolism. The P-body impairment by PQE Atx2 aggregates may represent a potential perspective for better understanding the pathogenesis of SCA2 and other neurodegenerative diseases.

## Results

### Atx2 sequesters DDX6 into aggregates

**Figure 1:**
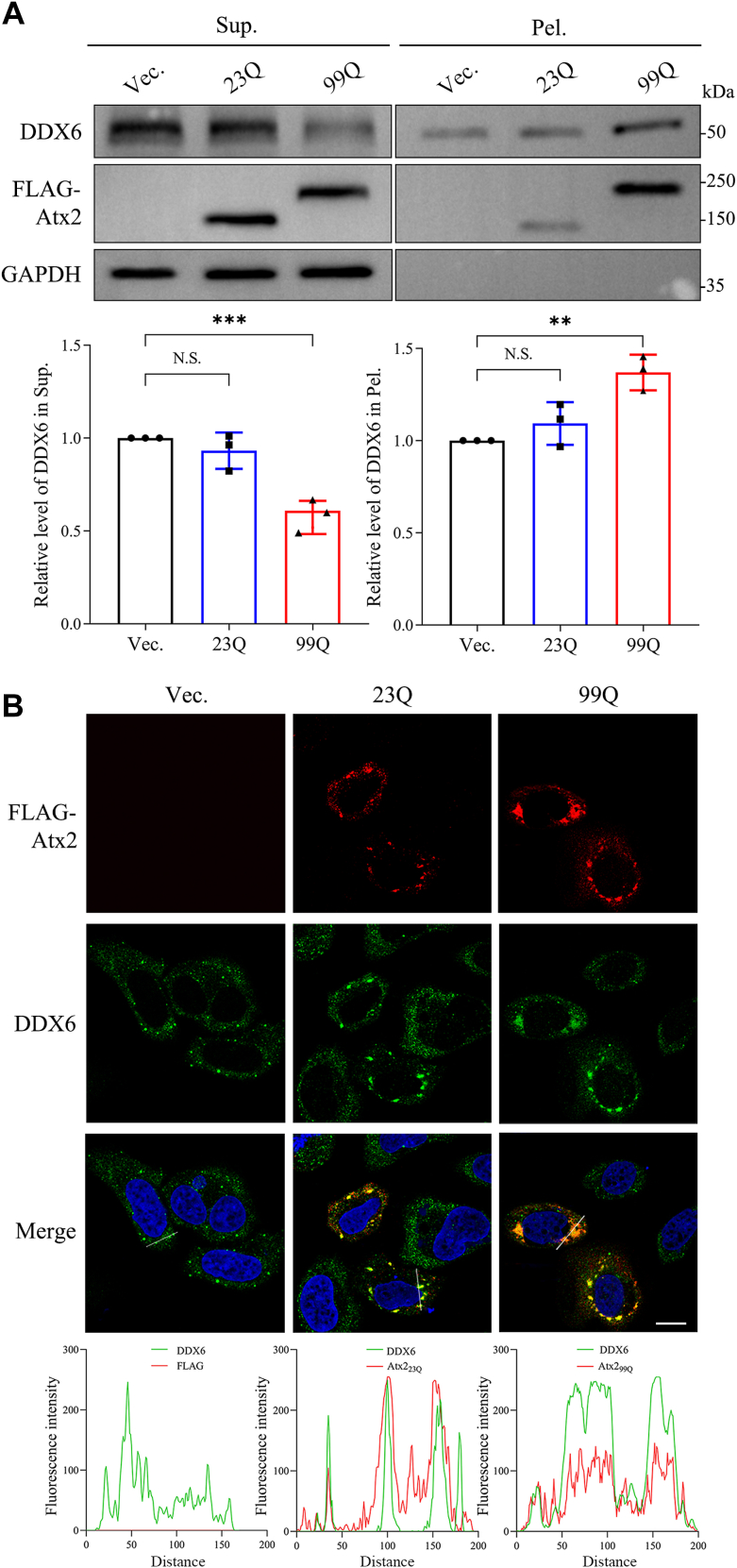
**Sequestration of endogenous DDX6 by PQE Atx2 in HeLa cells.***A*, S/P fractionation for characterizing sequestration of endogenous DDX6 into Atx2 aggregates. HeLa cells were transfected with each indicated plasmid, and after cultured for 48 h the cells were collected and lysed. The cell lysates were then subjected to S/P fractionation with Western blotting analysis. The data were obtained by normalizing to that of the vector control. Vec., vector; 23Q, Atx2_23Q_; 99Q, Atx2_99Q_; Sup., supernatant; Pel., pellet. Data are shown as Mean ± SD (n = 3). ∗∗, *p* < 0.01; ∗∗∗, *p* < 0.001; N.S., no significance. *B*, immunofluorescence imaging for co-localization of DDX6 with Atx2. HeLa cells were transfected with each indicated plasmid, and after cultured for 48 h the cells were fixed and immunostained with indicated antibodies. Atx2 was stained with anti-FLAG antibody (*red*), DDX6 was stained with anti-DDX6 antibody (*green*), and nuclei were stained with Hoechst (*blue*). Scale bar = 10 μm. *Bottom:* co-localization analysis of the fluorescence signals for the distance represented by white lines.

It has been reported that polyQ expansion of Atx2 with different lengths can cause different diseases, such as SCA2 (26, 28) and ALS (29), but the pathological mechanism is still poorly understood. To explore the impact of polyQ expansion on cellular homeostasis, we focused on the P-bodies that may be potentially influenced by PQE Atx2 aggregation. We firstly transfected normal (23Q) or PQE (99Q) Atx2 species (an alternative form with 1153 residues, Id: NP_002964.4) into HeLa cells and performed supernatant/pellet (S/P) fractionation experiment to separate the supernatant and pellet fractions of Atx2 and the endogenous DDX6 protein. The data showed that overexpression of Atx2_99Q_ could cause a significant increase of DDX6 in the pellet fraction and a decrease in the supernatant (Fig. 1*A*), but Atx2_23Q_ could not. It suggests that Atx2_99Q_ co-precipitated with DDX6 in pellet more efficiently than Atx2_23Q_, because PQE Atx2 formed large amounts of insoluble aggregates, as compared with the normal polyQ form (Fig. 1*A*). A similar result that PQE Atx2 sequesters endogenous DDX6 into aggregates was also obtained in HEK 293T cells (Fig. S1*A*).

To validate the sequestration, we also visualized the cellular co-localization of PQE Atx2 and endogenous DDX6 by immunofluorescence imaging in either HeLa or HEK 293T cells. In the cells without overexpressing Atx2, the endogenous DDX6 was dispersed in the cytoplasm. Atx2_23Q_ could form some aggregate-like puncta, but its main part is presented in a dispersed localization. Only a few DDX6 was co-localized with the aggregated puncta formed by Atx2_23Q_ (Figs. 1*B* and S1*B*). However, Atx2_99Q_ formed numerous large puncta and most DDX6 was well co-localized with Atx2_99Q_ in the cytoplasmic aggregated puncta (Figs. 1*B* and S1*B*, right).

We also examined the sequestration of the exogenous DDX6 by Atx2 aggregation. Analogously, Atx2_99Q_ formed more insoluble aggregates and co-precipitated more DDX6 into the pellet fraction than Atx2_23Q_, when Atx2 and DDX6 were co-expressed exogenously in HeLa cells (Fig. S2*A*). Exogenous DDX6 was co-localized with the aggregated puncta formed by both Atx2_23Q_ and Atx2_99Q_, but more DDX6 was sequestered by the Atx2_99Q_ aggregates because Atx2_99Q_ readily formed aggregated puncta in the cytoplasm (Fig. S2*B*, right). Overall, normal Atx2 partially forms aggregated puncta, while polyQ expansion may enhance its aggregation propensity. The Atx2 aggregates can sequester DDX6 into cytoplasmic aggregates or puncta.

### The N-terminal fragments of PQE Atx2 can also sequester DDX6

Through exogenous expression of a series of PQE Atx2, we observed occasionally that, in the pellet fraction, the PQE Atx2 protein could be degraded into two relatively stable fragments of ∼65-kDa and ∼45-kDa sizes (Fig. S3*A*), while the amount of the 45-kDa fragment increased with the polyQ expansion. According to the domain architecture of Atx2 (Fig. S3*B*), we assumed two cleavage sites possibly around the residues at 317 and 217 respectively. Thus, we constructed two N-terminal truncations of PQE Atx2 (Atx2_96Q_-N317, residues 1–317; Atx2_97Q_-N217, residues 1–217) for molecular markers, then we transfected the full-length Atx2_99Q_ as well as the two truncations respectively into HEK 293T cells and performed S/P fractionation to detect the degradation products in the pellet fraction. It showed that the molecular sizes of these fragments corresponded to those of the two truncations (Fig. S3*C*). To further confirm the degradation pattern of PQE Atx2, we constructed two stably expressing cell lines, pCDH-Atx2_23Q,_ and pCDH-Atx2_99Q_. With the cell culture progressing, Atx2_99Q_ was degraded mainly into the 65-kDa fragment and its content in the pellet fraction increased gradually (Fig. S3*E*), while that of Atx2_23Q_ remained at a relatively low level (Fig. S3*D*). This suggests that PQE Atx2 tends to be degraded into small N-terminal fragments in cells, which are relatively stable and remain in the aggregated forms.

**Figure 2:**
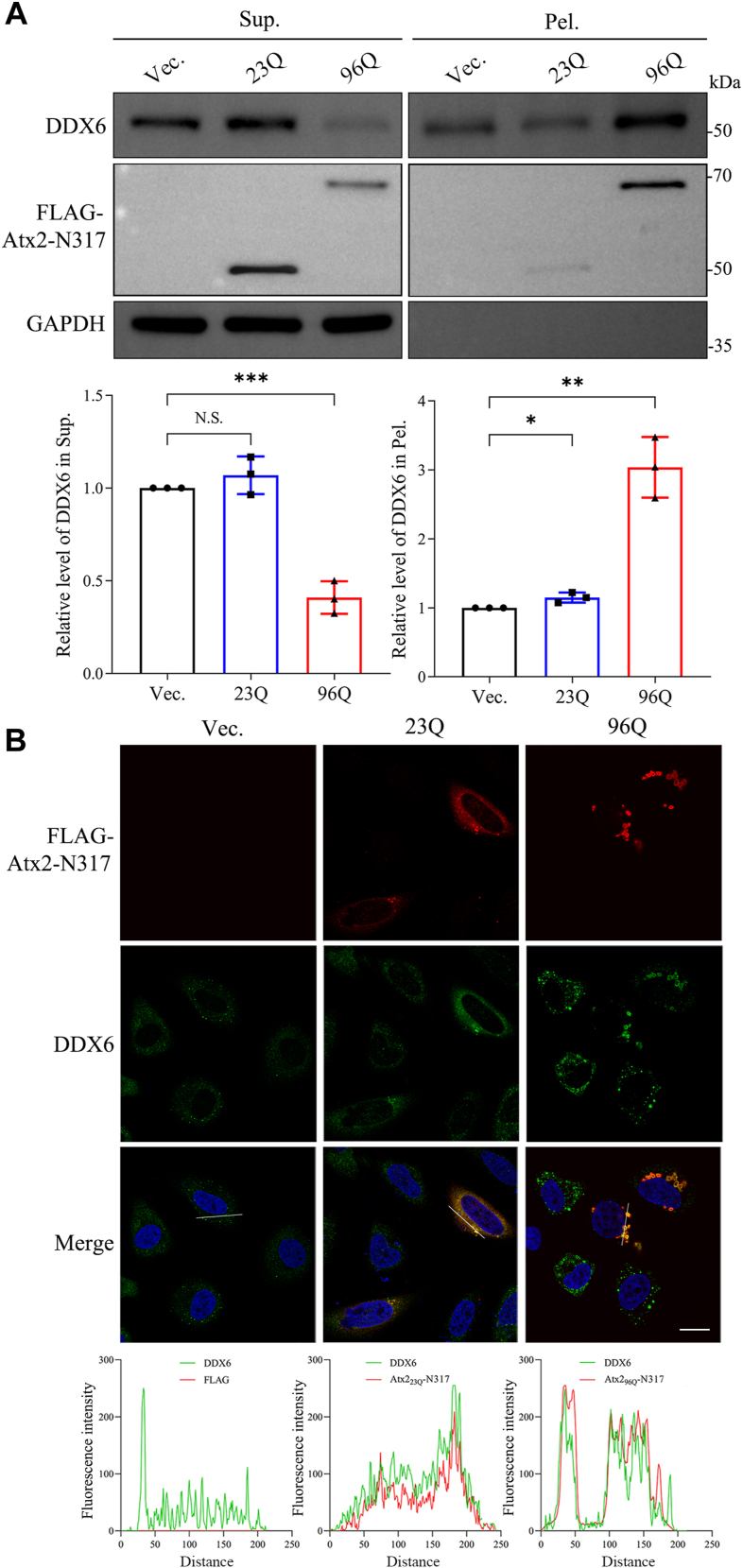
**Sequestration of endogenous DDX6 by PQE Atx2-N317 in HeLa cells.***A*, S/P fractionation for characterizing sequestration of endogenous DDX6 into Atx2-N317 aggregates. HeLa cells were transfected with each indicated plasmid and further cultured for 48 h. The lysates were subjected to S/P fractionation with Western blotting analysis. Vec., vector; 23Q, Atx2_23Q_-N317; 96Q, Atx2_96Q_-N317. Data are shown as Mean ± SD (n = 3). ∗, *p* < 0.05; ∗∗, *p* < 0.01; ∗∗∗, *p* < 0.001; N.S., no significance. *B*, immunofluorescence imaging for co-localization of DDX6 with Atx2-N317. HeLa cells were transfected with each indicated plasmid, and after 48-h culture, the cells were fixed and immunostained with indicated antibodies. Atx2-N317 was stained with anti-FLAG antibody (*red*), DDX6 was stained with anti-DDX6 antibody (*green*), and nuclei were stained with Hoechst (*blue*). Scale bar = 10 μm. *Bottom:* co-localization analysis of the fluorescence signals for the distance represented by white lines.

Since PQE Atx2 can be degraded into the N-terminal fragments and the polyQ-containing fragments easily accumulate in the cytoplasm, we then tested whether Atx2-N317 could interact with and sequester DDX6. Expectedly, as full-length Atx2_99Q_, Atx2_96Q_-N317 could significantly co-precipitate endogenous DDX6 into the pellet fraction both in HeLa cells (Fig. 2*A*) and HEK 293T cells (Fig. S4*A*), while a considerable amount of the Atx2_96Q_-N317 aggregates was also detected in the pellet fraction. However, Atx2_23Q_-N317 could only slightly increase the amount of DDX6 in the pellet fraction as in the case of full-length Atx2_23Q_, possibly due to insufficient aggregates formed by these Atx2_23Q_ species. Then we visualized cellular localizations of DDX6 and Atx2-N317 by immunofluorescence imaging. It exhibited that, unlike full-length Atx2_23Q_, most Atx2_23Q_-N317 protein was dispersed in the cytoplasm only with a few aggregated puncta. Atx2_96Q_-N317 could form numerous aggregated puncta entirely in the cytoplasm and well co-localize with endogenous DDX6 in the punctum foci (Figs. 2*B* and S4*B*, right).

We also exogenously co-expressed PQE Atx2-N317 and DDX6 in HeLa cells. A similar result was obtained showing that Atx2_96Q_-N317 formed large amounts of aggregates and remarkably sequestered exogenous DDX6 into aggregates (Fig. S5*A*), whereas Atx2_23Q_-N317 could also sequester overexpressed DDX6 but to a lesser extent. Furthermore, exogenously expressed DDX6 was co-localized with the cytoplasmic aggregated puncta formed by Atx2_96Q_-N317, while it was not co-localized with Atx2_23Q_-N317 due to few puncta formed (Fig. S5*B*).

Taken together, PQE Atx2 interacts with and sequesters DDX6 *via* its N-terminal portion and this fragment significantly aggravates the sequestration and co-localization effects, probably through its PQE tract for aggregation and LSm and LSmAD domains for interacting with DDX6.

### Characterization of the interaction between Atx2 and DDX6

**Figure 3:**
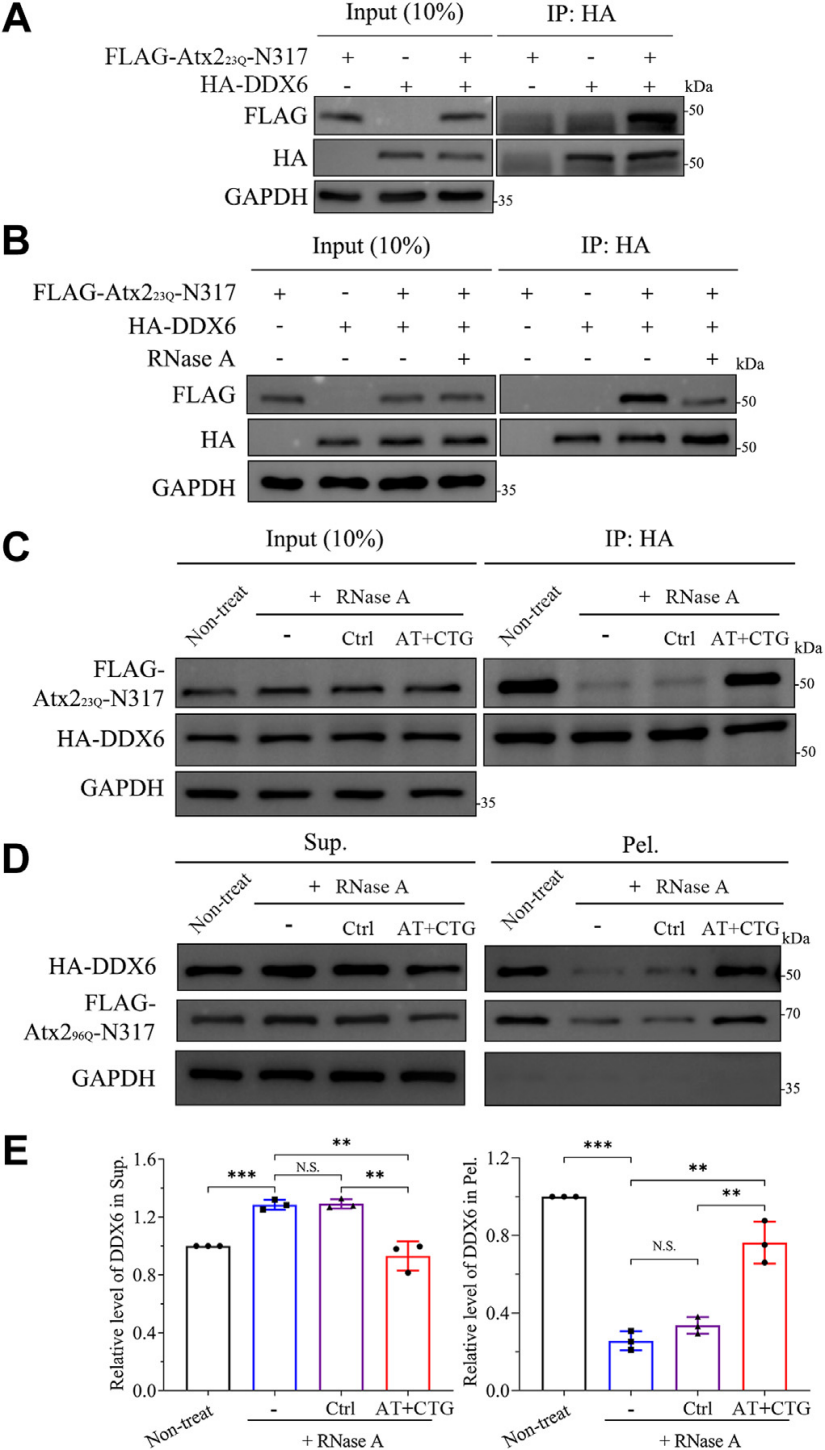
**Characterization of the interaction and sequestration between Atx2 and DDX6.***A*, Co-IP experiment examining the interaction between Atx2_23Q_-N317 and DDX6. HEK 293 T cells were co-transfected with each indicated plasmid and further cultured for 48 h, then the lysates were subjected to co-IP assay with anti-HA agarose beads. *B*, Co-IP experiment examining the interaction between Atx2_23Q_-N317 and DDX6 under the condition of RNase-A treatment. The lysates were subjected to co-IP assay with anti-HA agarose beads as (*A*) but with RNase-A treatment. *C*, Co-IP experiment examining the effects of RNase-A and ssDNA treatments on the interaction between Atx2_23Q_-N317 and DDX6. The treatments include non-treat, RNase-A treatment, and RNase-A plus ssDNA treatments. AT+CTG, (AT_5_)_5_+(CTG)_15_; Ctrl, control ssDNA with scrambled sequence. *D*, S/P fractionation for characterizing the effects of RNase A and ssDNA treatments on the sequestration of HA-DDX6 by FLAG-Atx2_96Q_-N317. HEK 293T cells were transfected with each indicated plasmid and cultured for 48 h. The cell lysates were treated with RNase A and ssDNA and then subjected to S/P fractionation with Western blotting. *E*, quantification of the DDX6 amounts in the supernatant and pellet fractions in (*D*). Data are shown as Mean ± SD (n = 3). ∗∗, *p* < 0.01; ∗∗∗, *p* < 0.001, N.S., no significance.

As PQE Atx2 and its N-terminal fragment can sequester DDX6 into aggregates, we wondered whether the sequestration process is mediated by a direct interaction between N-terminal Atx2 and DDX6. Therefore, we performed a co-immunoprecipitation (co-IP) experiment on the N-terminal fragment of Atx2 with normal polyQ length. FLAG-tagged Atx2_23Q_-N317 and HA-tagged DDX6 were co-expressed in HEK 293T cells to characterize their interaction. As in the previous report (17), our co-IP assay showed that Atx2_23Q_-N317 retained the capability of interacting with DDX6 (Fig. 3*A*), indicating that Atx2 interacts with DDX6 *via* its N-terminal region, including the polyQ tract and the LSm and LSmAD domains.

To further identify the specific region of Atx2 interacting with DDX6, we constructed a series of N-terminal fragments of Atx2, including Atx2-N184, Atx2-(29-104) (without polyQ tract), Atx2-(82-184) (LSm) and Atx2-(185-317) (LSmAD) (Fig. S6*A*), and performed co-IP assays to examine their interaction with DDX6. The data showed that Atx2-N184 and Atx2-(82-184), but neither Atx2-(29-104) nor Atx2-(185-317), could interact with DDX6 (Fig. S6, *B*–*E*). As for the DDX6 side, it is mainly comprised of two tandemly repeated RecA-like domains (RecA1 and RecA2), which possibly possess RNA-binding abilities (48, 49, 50). Our co-IP experiment showed that the RecA2 fragment but not the RecA1 in DDX6 was responsible for interacting with Atx2_23Q_-N317 (Fig. S7), further indicating that RNA binding contributes to the RBP association and sequestration. This implies that the two unique RNA-related regions, the LSm domain of Atx2 and the C-terminal helicase domain of DDX6, are essential to interacting with DDX6.

### Atx2 interacts with and sequesters DDX6 through RNA

Since the LSm domain of Atx2 and the C-terminal helicase domain of DDX6 are responsible for RNA binding involved in many aspects of RNA metabolism, we presumed that the interaction between Atx2 and DDX6 might be mediated by the RNAs with particular sequences. To examine the role of RNA in the association of Atx2 with DDX6, we co-transfected with FLAG-tagged Atx2_23Q_-N317 and HA-tagged DDX6 in HEK 293T cells and treated the lysates with RNase A and/or single-stranded DNA (ssDNA), followed by co-IP assay as described previously (51, 52). Obviously, in the absence of RNase A, a clear DDX6 band was observed in the gel, indicating that Atx2-N317 is associated with DDX6 in cells (Fig. 3*A*). When the cell lysates were treated with RNase A, the DDX6 band was remarkably attenuated (Fig. 3*B*). However, this band could be recovered considerably by the addition of an ssDNA chimera namely (CTG)_15_+(AT_5_)_5_ (Table S3) (Fig. 3*C*), but partially by (CTG)_15_ or (AT_5_)_5_ (Fig. S8*A*), as compared with the negative controls including scrambled ssDNA and double-stranded DNA (dsDNA) (Fig. S8*C*). It indicates that the association of Atx2 with DDX6 is mediated by some RNA sequences (*e.g.*, AU- and CUG-rich). In other words, Atx2 interacts with DDX6 indirectly, and these RNAs play a key role in their association and sequestration of various RBPs in cells.

To verify this assumption, we performed an S/P fractionation experiment under the conditions of RNase A and/or ssDNA treatments. The result showed that RNase-A treatment significantly destroyed the sequestration of DDX6 by Atx2_96Q_-N317 (Fig. 3, *D* and *E*), while (AT_5_)_5_ and (CTG)_15_ could partially recover the disruptive effect of RNase A and correspondingly the soluble amount in the supernatant was reduced (Fig. S8*B*). Intriguingly, the chimeric ssDNA (CTG)_15_+(AT_5_)_5_ could recover the sequestration almost completely (Fig. 3, *D* and *E*), whereas the scrambled ssDNA and dsDNA, as controls, almost lost their rescuing effects (Fig. S8*D*). This implies that the sequestration of DDX6 by the Atx2_96Q_-N317 aggregates is dependent on their association mediated by some RNA sequences.

### PQE Atx2 aggravates mis-splicing of pre-mRNA through sequestering DDX6

**Figure 4:**
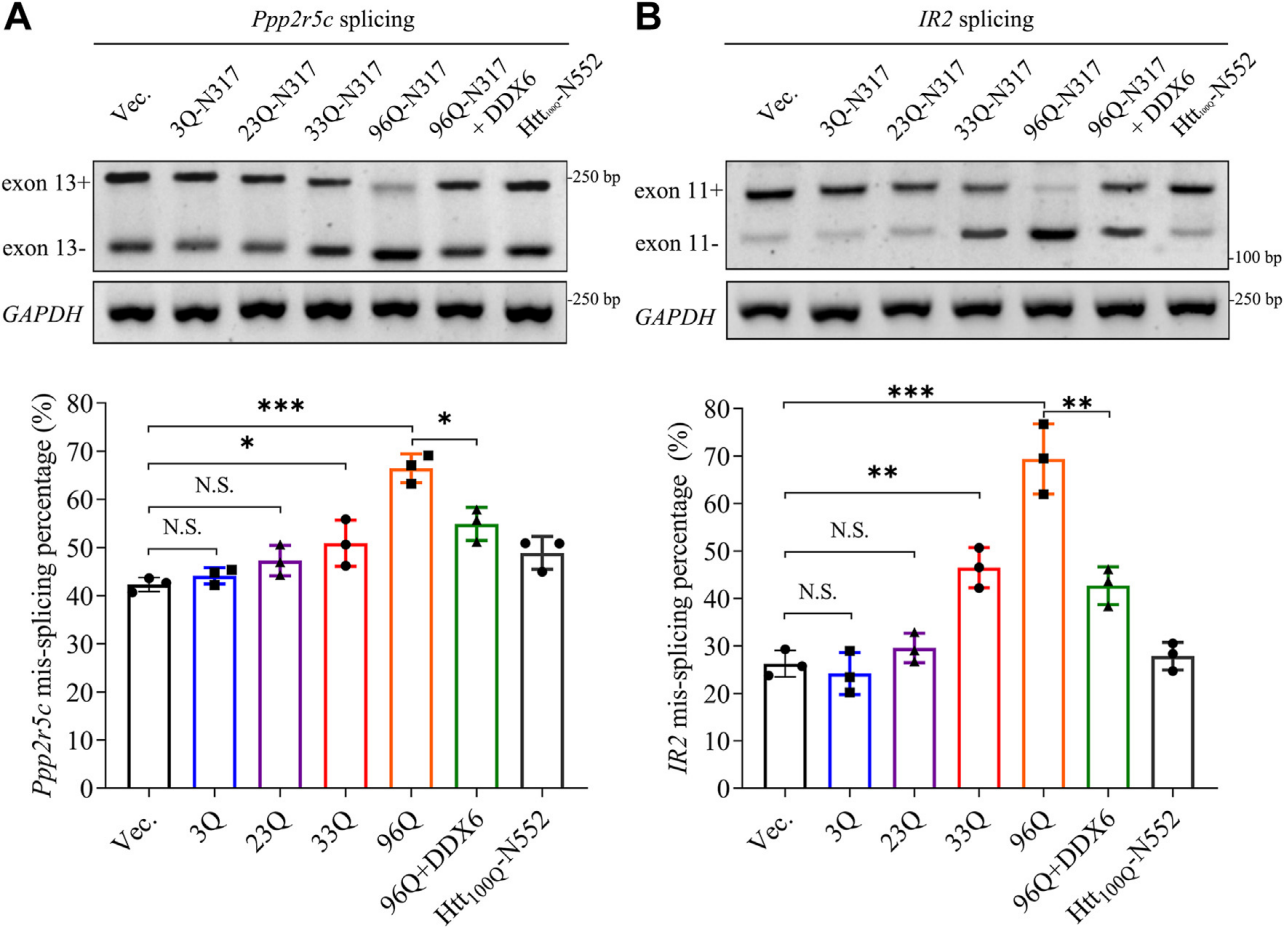
**Atx2 aggregation aggravates the mis-splicing of *Ppp2r5c* and *IR2* through sequestering DDX6.***A*, effect of Atx2-N317 aggregation on the mis-splicing of *Ppp2r5c* pre-mRNA. HEK 293T cells were transfected with each indicated plasmid and collected after 48-h culture, and then the reversely transcribed cDNAs were subjected to semi-quantitative PCR to amplify the *Ppp2r5c* amplicons either lacking (*bottom*) or retaining exon 13 (*top*). The percentage of *Ppp2r5c* mis-splicing was estimated by the formula: P (%) = exon13^-^/(exon13^-^ + exon13^+^). *B*, effect of Atx2-N317 aggregation on the mis-splicing of *IR2* pre-mRNA. The percentage of *IR2* mis-splicing was estimated by the formula: P (%) = exon11^−^/(exon11^−^ + exon11^+^). Data are shown as Mean ± SD (n = 3). ∗, *p* < 0.05; ∗∗, *p* < 0.01; ∗∗∗, *p* < 0.001; N.S., no significance.

Since PQE Atx2 could sequester DDX6 into aggregates, we then asked whether this sequestration has an impact on RNA metabolism. As reported, many mRNAs exhibit an increased mis-splicing under pathological conditions (53), while the RNA helicase DDX6 can relieve this mis-splicing event, for example, overexpression of DDX6 can reduce the mis-splicing of *Ppp2r5c* and *IR2* in the patients of myotonic dystrophy type 1 (MD1) (54). We speculated that PQE Atx2-N317 aggregation might aggravate the mis-splicing event in cells through sequestration of DDX6. To test this hypothesis, we extracted RNAs from the HEK 293T cells transfected with either normal or PQE Atx2-N317 and performed RT-PCR to semi-quantify the mis-splicing levels of *Ppp2r5c* and *IR2* pre-mRNAs. The result showed that the mis-splicing levels of *Ppp2r5c* and *IR2* remained almost unchanged in the cells transfected with Atx2_23Q_-N317 compared to the polyQ-deficient variant (3Q) (Fig. 4, *A* and *B*). However, it was significantly enhanced when the cells overexpressed Atx2_33Q_-N317 or Atx2_96Q_-N317 (Fig. 4, *A* and *B*), suggesting that PQE Atx2 aggregation aggravates the mis-splicing levels of *Ppp2r5c* and *IR2* pre-mRNAs. Thus, this mis-splicing event is presumably caused by dysregulation of nuclear splicing factors, such as muscleblind-like protein (MBNL1) (54), because shuttling of the splicing factors between cytoplasm and nucleus may be disturbed by sequestration of DDX6.

### PQE Atx2 aggregation reduces P-body numbers in cytoplasm

**Figure 5:**
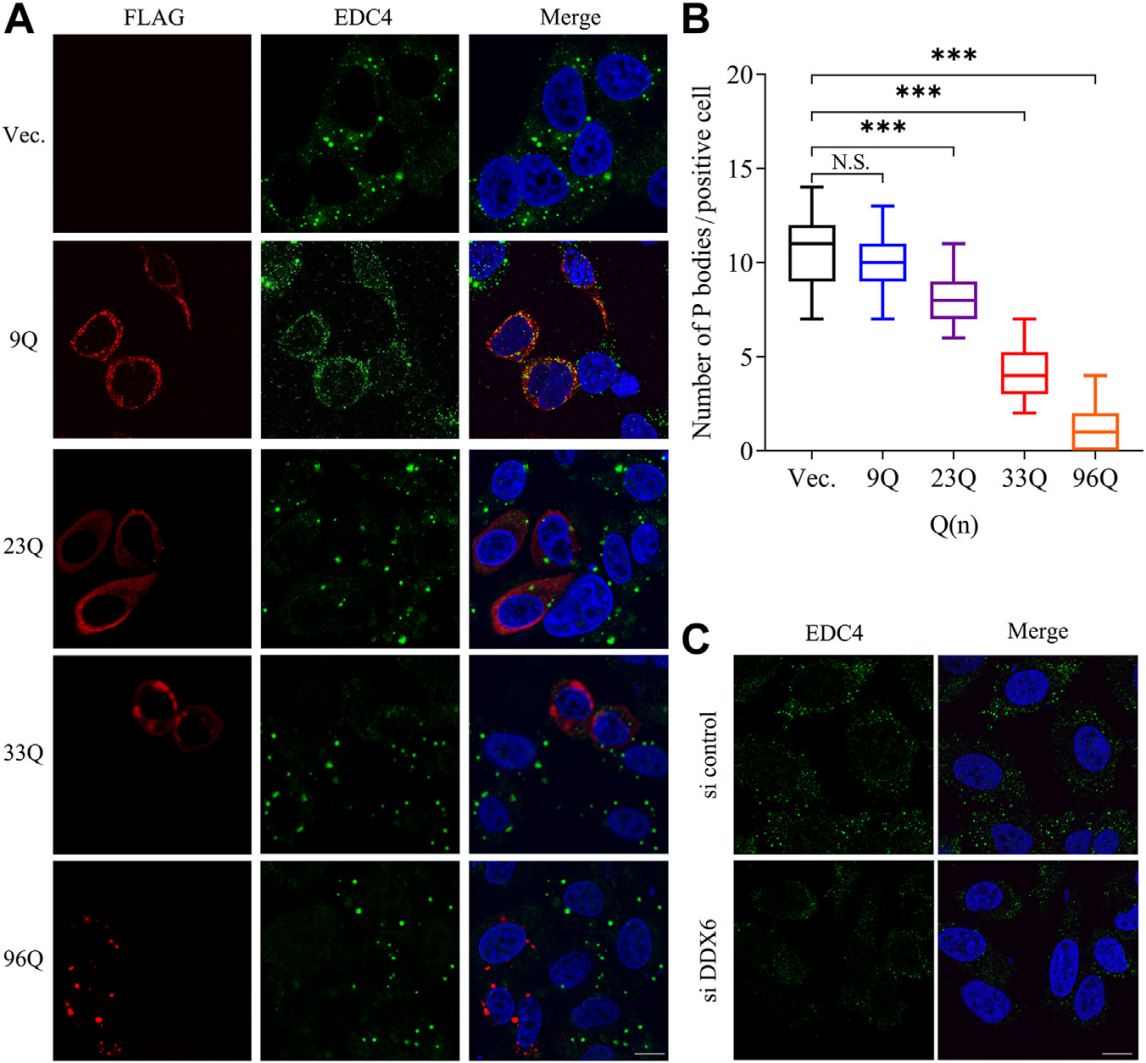
**Effects of Atx2 aggregation on the assembly of P-bodies.***A*, immunofluorescence imaging for P-body formation influenced by Atx2 aggregation. HeLa cells were transfected with Atx2-N317 with different polyQ lengths and collected after 48-h culture. The cells were fixed and immunostained with indicated antibodies. Atx2-N317 was stained with anti-FLAG antibody (*red*), EDC4 was stained with anti-EDC4 antibody (*green*) as a P-body marker, and nuclei were stained with Hoechst (*blue*). Scale bar = 10 μm. *B*, quantification of the P-body formation upon Atx2-N317 overexpression. The data were estimated from counting the P-body numbers in Atx2-N317 positive cells by using ImageJ software. Data are shown as Mean ± SD (n = 30). ∗∗∗, *p* < 0.001; N.S., no significance. *C*, immunofluorescence imaging for the P-body formation upon knockdown of endogenous DDX6 by siRNA. HeLa cells were transfected with DDX6 siRNA and collected after 48-h culture. EDC4 was stained with anti-EDC4 antibody (*green*) as a P-body marker, and nuclei were stained with Hoechst (*blue*). Scale bar = 10 μm.

It has been proposed that sequestration by protein aggregates has a considerable impact on the sequestered proteins (55), which is just like RNA interference to a target protein. P-bodies are a well-known class of cytoplasmic RNP granules, which are comprised of mRNAs complexed with proteins that play roles in translational repression including mRNA decapping and decay (44, 56, 57). While DDX6 has been reported to be essential for the P-body assembly (46), there is still no insight into which function is involved. As a previous report, P-bodies could form numerous dot-like puncta with high brightness in the cytoplasm under normal conditions as indicated by enhancer of mRNA decapping factor 4 (EDC4) (58) (Fig. 5*A*, first row), and depletion of DDX6 by RNA interference notably suppressed the formation of P-bodies (Fig. 5*C*). Therefore, we assessed the consequences of DDX6 sequestration by PQE Atx2 aggregates on the P-body assembly and distribution.

We transfected various Atx2-N317 species into HeLa cells, respectively, and visualized the cellular localization and distribution of P-bodies by immunofluorescence imaging. It exhibited that both Atx2_9Q_-N317 and Atx2_23Q_-N317 were dispersed in cytoplasm and the P-bodies were assembled and distributed well as normal (Fig 5*A*, second and third rows). On the contrary, the dot number of P-bodies reduced considerably in the cells overexpressing Atx2_33Q_-N317 (Fig. 5*A*, fourth row), while those disappeared almost completely upon overexpression of Atx2_96Q_-N317 (Fig. 5*A*, fifth row). We observed that, compared with Atx2_23Q_-N317, Atx2_33Q_-N317 could reduce the number of P-bodies by about 50% according to the statistics, while Atx2_96Q_-N317 could do over 90% (Fig. 5*B*). This result is consistent with the previous observation that overexpression of full-length Atx2 (79Q) dramatically reduced the number of P-bodies (17). It suggests that aggregation of PQE Atx2-N317 sequesters DDX6 into aggregates and reduces the functional availability of soluble DDX6 in cytoplasm, which thereby impairs the assembly of P-bodies directly.

### PQE Atx2 disrupts assembly of P-bodies by sequestering DDX6

**Figure 6:**
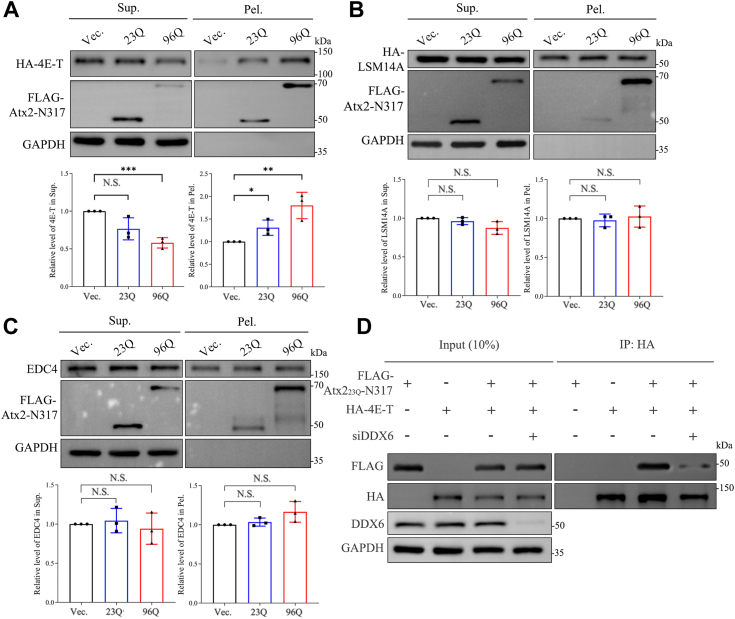
**PQE Atx2 aggregation disrupts the assembly of P-bodies by directly sequestering DDX6.***A, B, C*, S/P fractionation for characterizing sequestration of 4E-T (A), LSM14A (*B*), and EDC4 (*C*) by Atx2-N317. HEK 293T cells were transfected with each indicated plasmid and collected after 48-h culture, then the cell lysates were subjected to S/P fractionation with Western blotting analysis. Data are shown as Mean ± SD (n = 3). ∗, *p* < 0.05; ∗∗, *p* < 0.01; ∗∗∗, *p* < 0.001; N.S., no significance. (D) Co-IP experiment examining effect of DDX6 knockdown on the interaction between Atx2_23Q_-N317 and 4E-T. HEK 293T cells were co-transfected with FLAG-Atx2_23Q_-N317 and HA-4E-T with or without DDX6 siRNA, then the cell lysates were subjected to co-IP assay with anti-HA agarose beads.

Several P-body components have been shown to influence P-body assembly in mammalian cells, as their silencing leads to reduction or disappearance of the P-bodies (47). The P-body core components include DDX6 (59), eIF4E-tansporter (4E-T) (60), EDC4 (61), and Like Sm14 (LSM14A) (62). To examine whether PQE Atx2 aggregation sequesters other P-body components, we co-transfected PQE Atx2-N317 with 4E-T or LSM14A in HEK 293T cells and the cell lysates were subjected to S/P fractionation. The data showed that due to overexpression of Atx2-N317, the protein level of 4E-T in the pellet fraction increased remarkably (Fig. 6*A*), and the PQE form sequestered 4E-T more efficiently than the normal form. However, PQE Atx2-N317 did not sequester LSM14A (Fig. 6*B*) nor endogenous EDC4 (Fig. 6*C*) into the pellet fraction.

Because 4E-T can interact with DDX6 and LSM14A *via* its different domains (63), the association and sequestration of 4E-T by Atx2 might be mediated by DDX6. To confirm this hypothesis, we applied siDDX6 RNA interference and performed co-IP assay with co-transfected FLAG-tagged Atx2_23Q_-N317 and HA-tagged 4E-T in HEK 293T cells. The result showed that Atx2_23Q_-N317 could associate with 4E-T, while the knockdown of DDX6 attenuated the association between Atx2_23Q_-N317 and 4E-T (Fig. 6*D*). This indicates that Atx2 interacts with 4E-T indirectly, while DDX6 acts as a mediator for their association. Taken together, PQE Atx2 aggregation impairs P-body assembly by interacting with and sequestering DDX6 and other P-body components in an indirect manner.

### PQE Atx2 aggregation promotes MARF1-mediated mRNA decay *via* disturbing the P-body assembly

**Figure 7:**
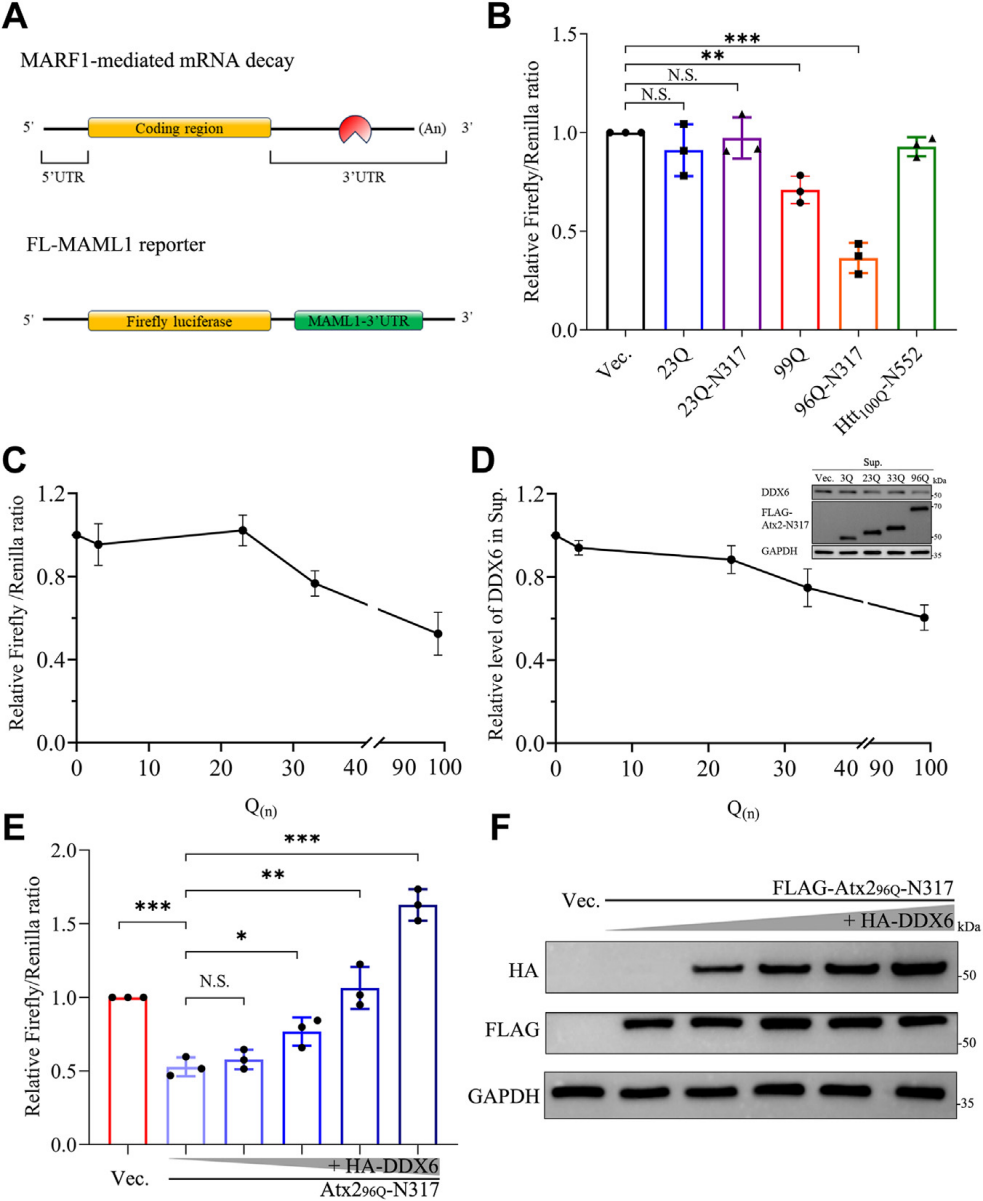
**PQE Atx2 aggregation promotes MARF1-mediated mRNA decay.***A*, schematic diagrams of MARF1-mediated mRNA decay (*top*) and the FL-MAML1 reporter (*bottom*). The reporter plasmid was constructed by linking the *MAML1* 3′UTR sequence to the pmirgol1 vector behind the *Firefly* luciferase element. *B*, FL/RL activity ratios upon overexpression of various PQE Atx2 variants. HEK 293T cells were transfected with each indicated plasmid and collected after 48-h culture. The fluorescence intensities were recorded and the relative FL/RL ratio was normalized to that of the vector. Data are shown as Mean ± SD (n = 3). ∗∗, *p* < 0.01; ∗∗∗, *p* < 0.001; N.S., no significance. *C*, effect of polyQ length in Atx2-N317 on the FL/RL activity ratio. (*D*) Effect of polyQ length in Atx2-N317 on the DDX6 level. A portion of the cell lysates in (*C*) was subjected to detection of the DDX6 level in the supernatant. *E*, rescuing effect of DDX6 overexpression on the luciferase activity suppressed by Atx2_96Q_-N317 aggregation. HEK 293T cells were co-transfected with FLAG-Atx2_96Q_-N317 and HA-DDX6 with an increasing dose, about 24 h later the reporter plasmid was transfected. After cultured for another 24 h, the cells were harvested and lysed, and then the lysates were subjected to detection of luciferase activity. Data are shown as Mean ± SD (n = 3). ∗, *p* < 0.05; ∗∗, *p* < 0.01; ∗∗∗, *p* < 0.001; N.S., no significance. *F*, Western blotting analysis for DDX6 in the supernatant fraction for the samples from (*E*).

Since PQE Atx2 aggregation impairs P-body assembly by sequestering DDX6 and other components, we thus enquired whether this sequestration process influences the function of DDX6 in RNA metabolism. Mammalian MARF1 is an endoribonuclease that is associated with P-bodies in an antagonized state (58, 64). In general, MARF1 binds to the 3′UTR of targeted mRNAs and facilitates their decay (Fig. 7*A*) (65), while P-bodies can inhibit MARF1-mediated mRNA decay directly *via* retaining MARF1 in a suppressed state (64). As PQE Atx2 can sequester DDX6 into cytoplasmic aggregates and impair the assembly of P-bodies, we set out to determine whether the loss of P-bodies affects the MARF1 activity by using the dual-fluorescence technique (64). We firstly constructed a reporter plasmid namely FL-MAML1 (Fig. 7*A*), which contains the *Renilla* luciferase (RL) gene for a basal expression standard and the *Firefly* luciferase (FL) gene combining with the 3′UTR of *MAML1*, one of the target genes of MARF1 (58). To validate the reporter, we conducted knockdown or overexpression of DDX6 in HEK 293T cells and measured their FL/RL ratios. When the cells were treated with siDDX6 RNA, the FL/RL ratio decreased significantly by about 80% (Fig. S9*A*); whereas the ratio increased by over 100% upon overexpression of DDX6 (Fig. S9*B*). It indicates that the dual-fluorescence reporter is appropriate to respond to the MARF1 activity influenced by fluctuation of the DDX6 level.

Then we co-transfected the FL-MAML1 reporter with various Atx2 species (Atx2_23Q_, Atx2_99Q_, Atx2_23Q_-N317, Atx2_96Q_-N317) or the N-terminal fragment of huntingtin (Htt) (Htt_100Q_-N552) in HEK 293T cells respectively, and measured and compared their FL/RL ratios. As a result, both Atx2_99Q_ and Atx2_96Q_-N317 could efficiently reduce the FL/RL ratios, suggesting that disruption of the P-bodies released the MARF1 endoribonuclease and hence enhanced degradation of the FL-MAML1 reporter (Fig. 7*B*). Notably, the MARF1 activity was enhanced by Atx2_96Q_-N317 more obviously than that by full-length Atx2_99Q_, possibly because the PQE Atx2 fragment was more aggregation-prone than the full-length form. However, the 23Q forms of Atx2 and its fragment had only little effect on the FL/RL ratio. We also set Htt-N552 as a control (66, 67), in which overexpression of Htt_100Q_-N552 did not affect the MARF1 activity at all (Fig. 7*B*). It implies that activation of the MARF1 activity is a specific event depending on PQE Atx2 aggregation and sequestration. We then enquired about the effect of polyQ length on the MARF1 activity recovered from P-body suppression. We co-transfected the reporter with Atx2-N317 of various polyQ lengths (3Q, 23Q, 33Q, 96Q) respectively in cells. The FL/RL ratio decreased gradually when overexpression of the Atx2-N317 species with their polyQ lengths larger than the threshold (23Q) (Fig. 7*C*), indicating that enhancement of the MARF1 activity is dependent on the polyQ length. Simultaneously, the amount of the soluble DDX6 protein also decreased as polyQ expansion (Fig. 7*D*). It demonstrates that enhancement of the MARF1 activity by PQE Atx2 aggregation is caused by sequestering DDX6 and disrupting the P-body assembly, leading to MARF1 release from the P-bodies to degrade mRNA in the cytoplasm.

To further confirm the above observation that the reduction of FL/RL ratio is really caused by a change of the DDX6 partitioning in the cytoplasm, we carried out a DDX6 rescue experiment on the suppressing effect of PQE Atx2. PQE Atx2-N317 aggregation was proposed to sequester DDX6 and reduce the availability of soluble DDX6 that functions in the P-body assembly. The P-body impairment may cause the release of MARF1 which facilitates the decay of the downstream mRNA. We transferred DDX6 with an increasing dose in the cells overexpressing Atx2_96Q_-N317 and recorded the FL/RL ratios. As the above observation, Atx2_96Q_-N317 could reduce the FL/RL ratio by about 50%. With the increase of DDX6, the FL/RL ratio increased significantly in a dose-dependent manner (Fig. 7*E*), indicating that the suppressing effect caused by Atx2_96Q_-N317 was gradually recovered by DDX6. At the same time, the amount of soluble DDX6 fraction was also raised with the increase of DDX6 expression (Fig. 7*F*). These results indicate that the enhancement of mRNA decay is just caused by sequestration of the soluble DDX6 that leads to P-body disassembly and release of MARF1 for endoribonuclease activity.

### PQE Atx2-N317 reduces MARF1-targeted mRNA levels and leads to translational repression

**Figure 8:**
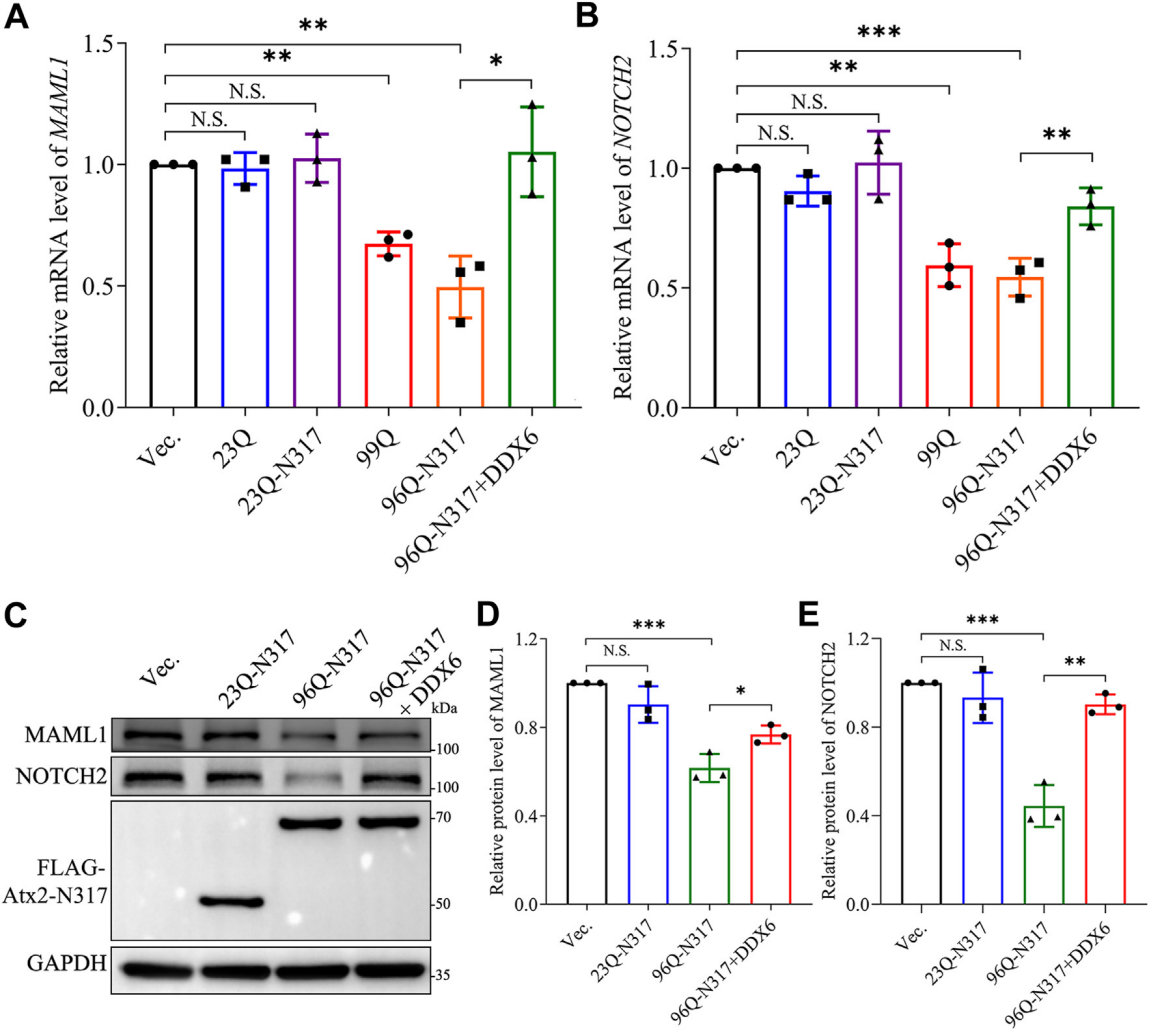
**PQE Atx2-N317 reduces the MARF1-targeted mRNA level leading to their translational repression.***A, B*, detection of the *MAML1* and *NOTCH2* mRNA levels in the cells overexpressing various PQE Atx2 variants. HEK293T cells were transfected with each indicated plasmid and collected after 48-h culture. The cell lysates were subjected to reverse transcription into cDNA and then quantitative PCR for *MAML1* (*A*) and *NOTCH2* (*B*). The target mRNA levels were normalized to the mRNA level of *GAPDH* and set the vector as a control. Data are shown as Mean ± SD (n = 3). ∗, *p* < 0.05; ∗∗, *p* < 0.01; ∗∗∗, *p* < 0.001; N.S., no significance. 23Q, Atx2_23Q_; 99Q, Atx2_99Q_; 23Q-N317, Atx2_23Q_-N317; 96Q-N317, Atx2_96Q_-N317. *C*, Western blotting analysis for endogenous MAML1 and NOTCH2 proteins in the supernatant fraction expressing various PQE Atx2-N317 proteins. HEK 293T cells were transfected with each indicated plasmid and subjected to Western blotting for analyzing the MAML1 and NOTCH2 levels in the supernatant fraction. *D, E*, quantitative analysis of the protein levels of MAML1 and NOTCH2 in the supernatant fraction for the samples from (*C*). Data are shown as Mean ± SD (n = 3). ∗, *p* < 0.05; ∗∗, *p* < 0.01; ∗∗∗, *p* < 0.001; N.S., no significance.

Based on the exogenous dual-fluorescence reporter for the *MAML1* mRNA decay driven by the MARF1 endoribonuclease, we revealed that PQE Atx2 aggregation promotes MARF1-mediated mRNA decay by sequestering DDX6. We then further assessed other MARF1-targeted mRNAs by quantitative RT-PCR (RT-qPCR) as described previously (58). As a result, the mRNA levels of *MAML1* and *NOTCH*2 were significantly decreased in the cells overexpressing Atx2_99Q_ or Atx2_96Q_-N317, while the addition of DDX6 could rescue the decreasing effect (Fig. 8, *A* and *B*). Similar results were also obtained that the mRNA levels of *IGF2BP1* and *ATXN7L3* genes were also reduced by Atx2_99Q_ or Atx2_96Q_-N317, and in turn recovered by addition of DDX6 (Fig. S10, *A* and *B*).

Next, we detected the protein levels of endogenous MAML1 and NOTCH2 upon overexpression of Atx2-N317 by Western blotting. It showed that the protein levels of MAML1 and NOTCH2 were significantly reduced in the cells overexpressing Atx2_96Q_-N317 but not in the normal polyQ form (Fig. 8, *C*–*E*). Moreover, this reducing effect on the MARF1-targeted gene products could be restored by the addition of DDX6. This result from the protein level is consistent with the above observation that PQE Atx2 can reduce the MARF1-targeted mRNA level. Together, PQE Atx2-N317 aggregation and sequestration of DDX6 downregulate the MARF1-targeted mRNA levels, leading to their translational repression.

## Discussion

**Figure 9:**
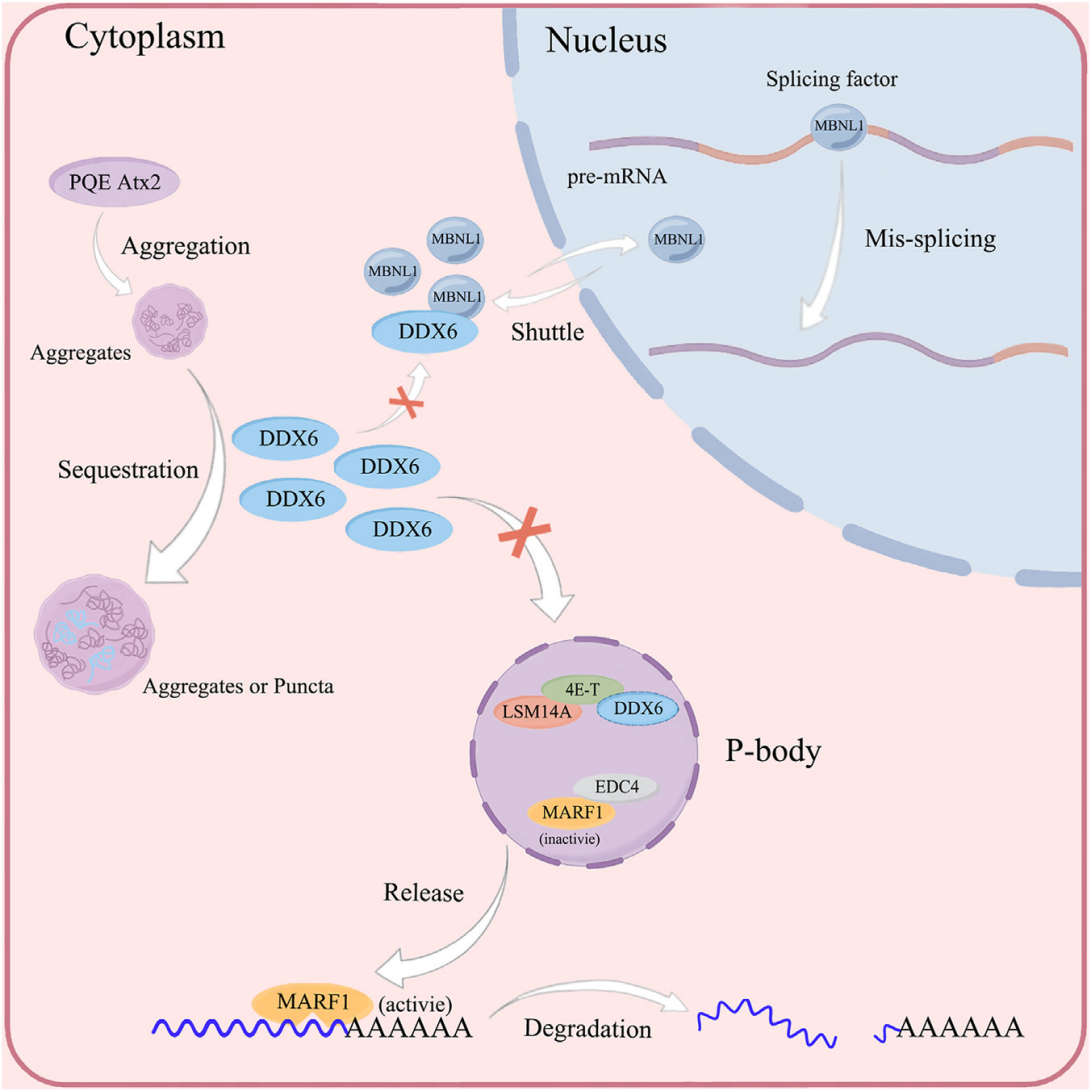
**Consequences of DDX6 sequestration by PQE Atx2 aggregates: impairment of cellular P-body homeostasis and aggravation of pre-mRNA mis-splicing.** PQE Atx2 forms aggregates and sequesters DDX6 into aggregated puncta in the cytoplasm. Depletion of the soluble DDX6 impairs the assembly of cellular P-bodies and triggers the release of the endoribonuclease MARF1, which leads to robust reduction of the MARF1-targeted mRNA levels and translational repression of the expressed proteins. On the other hand, depletion of cytoplasmic DDX6 may also interfere with the shuttle of splicing factors (such as MBNL1) into nucleus, which aggravates mis-splicing of the pre-mRNAs.

We have found that PQE Atx2 aggregation sequesters DDX6 into cytoplasmic aggregates or puncta, impairs the cellular P-body homeostasis, and aggravates the pre-mRNA mis-splicing (Fig. 9). Disruption of the P-bodies may consequently release the endoribonuclease MARF1 to the cytoplasm, leading to downregulation of the MARF1-targeted mRNA levels and translational repression of the expressed proteins. Thus, sequestration of DDX6 by PQE Atx2 aggregates could be considered as an alternative pathomechanism for SCA2 and other neurodegenerative diseases (68).

Note that, in this study, we have applied an overexpression system to elucidate the molecular mechanism underlying PQE Atx2 aggregation and sequestration of DDX6, which may inevitably have some weakness or limitation due to the potential impact of high concentration on protein aggregation. However, Atx2 aggregation may predominantly depend on the length of its polyQ tract. We have also constructed polyQ-deficient Atx2 variants and observed that, compared with normal (Atx2_23Q_) and its PQE variants (Atx2_33Q_, Atx2_99Q_), nearly all the Atx2_9Q_ molecules are retained in the supernatant fraction, but only a few aggregates can be detected in the pellet (Fig. S3*A*). This suggests that the polyQ-tract length is more important than protein concentration in driving Atx2 aggregation and sequestration.

### Atx2 can be degraded into N-terminal fragments in cells

As known, PQE Atx2 is prone to aggregation and the insoluble aggregates accumulate in the cytoplasm. Interestingly, we have observed two small-sized N-terminal fragments, ∼65 kDa and ∼45 kDa respectively, in the pellet fraction. The 45-kDa fragment probably originated from further degradation of the 65-kDa one, and the amounts of these fragments in the pellet increase with the polyQ expansion. Thus, we deduce that full-length Atx2 may harbor two potential cleavage sites at residues 317 and 217, respectively. As reported (69), the full-length Atx2 with either normal (*e.g.*, 23Q) or PQE (*e.g.*, 99Q) may experience proteolysis resulting in the polyQ-containing N-terminal fragments. The fragments with normal polyQ will be cleared by cellular proteases in time. However, the aggregates formed by the PQE Atx2 fragments are hard to further degrade efficiently due to their insolubility and proteolytic inaccessibility. Thus, the N-terminal fragments of PQE Atx2 are prone to aggregation and readily accumulate in the cytoplasm.

Protein fragmentation is a common cellular event, reminiscent of the Exon 1 fragment of PQE Htt that exhibits a greater aggregation propensity and cytotoxicity, compared with the full length (70, 71). Under pathological conditions, the amyloidogenic proteins are readily degraded into smaller fragments of more cytotoxic and pathogenic, which may partly contribute to the pathogenesis of SCA2 and other diseases.

### The Atx2-DDX6 interaction and sequestration is mediated by RNA

DDX6 is a member of RNA helicase superfamily 2 and is highly conserved in eukaryotes from yeast to humans characterized by its DEAD motif (72, 73, 74). Both Atx2 and DDX6 are RBPs related to RNA, their association and sequestration may be mediated by RNA binding, as in the case of TDP-35 with TIA1 (51) and PABPN1 with CFIm25 (52). By combined treatment of RNase A and chimeric ssDNA, we have demonstrated that the RNAs with particular sequences play a crucial role in mediating the interaction between Atx2 with DDX6 as well as sequestration of DDX6 by the PQE Atx2 aggregates. We have also characterized that the RNA-binding regions in both RBPs actually act as the protein interacting sites, and sequestration of RBPs into aggregates is dependent on the RNA-mediated interactions between these RBPs.

### Sequestration of DDX6 leads to disruption of the P-body assembly

DDX6 is mainly involved in RNA metabolism in the cytoplasm functioning in alternative splicing, ribosome biogenesis, RNA transport, mRNA decay, and translation (48, 73). Our data have shown that, as RNA interference (54), sequestration of DDX6 by PQE Atx2 aggregates results in the mis-splicing of the target mRNAs indirectly (Figs 4 and 9). DDX6 is also a core component of P-bodies responsible for the P-body assembly and homeostasis (35, 63, 64), while silencing of DDX6 by RNA interference leads to a reduction of the P-body numbers (47). P-bodies are a well-known class of cytoplasmic RNP granules, which are comprised of RNA-binding proteins and their cognate mRNAs that mainly play roles in mRNA decay and translational repression (75). As shown, the formation of biomolecular assemblies, such as droplets and aggregates (32), is essential for the sequestration processes in cells (76). Either full-length Atx2_23Q_ or its N-terminal fragment (Atx2_23Q-_N317) can only form few foci-like puncta in the cytoplasm that fails to sequester endogenous DDX6 (Figs. 1 and 2), although they do interact with DDX6 *via* its LSm domain (Figs. 3 and S6). As other polyQ proteins, polyQ expansion in both the full length (Atx2_99Q_) and its fragment (Atx2_96Q_-N317) enhances self-aggregation of the proteins and drives sequestration of DDX6 into insoluble aggregates (Figs. 1 and 2). Moreover, fragmentation of PQE Atx2 facilitates protein aggregation and thereby sequestration of cytoplasmic DDX6 into the aggregates. These processes may exacerbate the impairment of P-body assembly, leading to upregulation of mRNA decay and translational repression (Figs. 7 and 8). Thus, dysregulation of RNA metabolism may provide an alternative perspective explaining why PQE Atx2 aggregation elevates the risk of neurodegenerative diseases (2).

A previous study showed that Atx2 interacts with DDX6 through its LSm and LSmAD domains in a yeast two-hybrid system and Atx2 overexpression exerts a noticeable effect on the P-body homeostasis (17). However, it also asserted that PQE Atx2 (79Q) does not aggregate into inclusions, but colocalizes with DDX6 in cellular structures under the condition of arsenite or heat-shock treatment. Moreover, the Atx2 paralog (Atx2L) was considered as a regulator of P-bodies and SGs, since Atx2L overexpression and reduction directly decreases the number and size of P-bodies in HeLa cells (18). In this study, we have further identified the LSm domain but not including LSmAD as the interaction sites on Atx2, and this unique interaction is mediated by RNA. PQE Atx2 and its N-terminal fragments can form insoluble aggregates and sequester DDX6 remarkably into the aggregates or puncta (Fig. 2*A*). Of note, this inconsistency may be originated from the different sequences of Atx2 we have applied in the N-terminus ahead the polyQ tract.

### P-body impairment upregulates MARF1-mediated mRNA decay

MARF1 is a mammalian endoribonuclease, an RBP that associates with P-bodies (65). It is generally considered an mRNA decay regulator that binds to the 3′UTR of targeted mRNAs and mediates their decay (58). In the presence of intact P-bodies, EDC4 binds to MARF1 leading to the recruitment of MARF1 into P-bodies that inhibits its RNA-binding and mRNA decay potentials (64). However, disruption of the P-body homeostasis by depletion of DDX6 results in releasing of MARF1 into the cytoplasm, in which MARF1 exists in an active state and robustly decays target mRNAs. In turn, our data substantiate that the P-bodies play a direct role in regulating MARF1-mediated mRNA decay by recruiting MARF1 into P-bodies and thus render MARF1 unable to interact with and degrade targeted transcripts.

### An alternative mechanism for protein-aggregation pathology

It is well-known that aggregation of proteins including polyQ proteins can cause neurodegenerative diseases. We have recently revealed that Atx2 can sequester Raptor into aggregates, and thereby impair cellular mTORC1 signaling and induce autophagy (77). The current study demonstrates that PQE Atx2 aggregation is deleterious to mRNA metabolism by sequestrating cytoplasmic DDX6 and impairing P-body assembly, whereas restoration of the P-body functionalities by addition of DDX6 may be a potential therapeutic approach for SCA2 as well as ALS.

Many RBPs and their bound RNAs are associated with the formation of SGs and P-bodies in cells (78, 79), in which mis-regulated processing of mRNA is also associated with the pathologies of neurodegenerative diseases (80). However, the central role of P-bodies in pathogenesis has not been established in other diseases.

In a model of Atx2-related diseases, PQE Atx2 can be degraded into more cytotoxic fragments, which readily aggregate and accumulate into insoluble inclusions during disease progression. The PQE Atx2 aggregates can sequester cytoplasmic DDX6 into the aggregates or inclusions by interacting with DDX6. This process may deprive the soluble DDX6 from functional availability and thereby impair the assembly of P-bodies. Normally, in the case of intact P-bodies, the EDC4-MARF1 interaction leads to the recruitment of MARF1 into the P-bodies and suppression of the MARF1-mediated mRNA decay in the cytoplasm. Thus, impairment of the mRNA homeostasis could be considered a common feature underlying the disease pathology, while targeting P-bodies might have broad therapeutic potentials for treatment (40). On the other hand, disruption of the P-bodies due to deprival of the soluble DDX6, MARF1 exists in an active state that can degrade the target mRNA, leading to dysregulation of mRNA and translational repression, which is considered as an alternative mechanism for Atx2-associated neurodegenerative diseases.

In all, this study provides further evidence for the previously proposed model that sequestration of cellular essential factors into aggregates or inclusions leads to depletion of the targeted proteins and consequently to proteinopathies in neurodegeneration (55, 81, 82, 83, 84). The sequestration model involving PQE Atx2 aggregation will represent a potential perspective for further elucidating the pathological mechanisms underlying SCA2 and other neurodegenerative diseases.

## Experimental procedures

### Plasmids, oligonucleotides, and antibodies

The DNA sequences encoding Atx2 and its variants with different lengths of Gln residues (9Q, 23Q, 33Q, 99Q) were cloned into a FLAG-pcDNA3.1 vector *via* BamH I/Not I sites, while the 3Q variant was cloned into the same vector *via* Not I/Xba I sites. For the N-terminal truncations, the cDNAs encoding the sequences of Atx2-N217 and Atx2-N317 were PCR amplified from the pcDNA3.1-FLAG-Atx2 plasmid using the primers of Atx2-F and Atx2-N217-R or Atx2-N317-R, and then the DNA fragments were inserted into the FLAG-pcDNA3.1 vector respectively *via* BamH I/Not I sites. It is noteworthy that the DNA sequence of Atx2 was adopted from the updated version (NM_002973.4), which has the N-terminal 160 residues shorter than the previous one (NM_002973.3). The cDNA encoding DDX6, DDX6-A1, DDX6-A2, 4E-T or LSM14A was cloned into an HA-pcDNA3.1 vector *via* BamH I/Not I. The 3′UTR region of *MAML1* was PCR amplified from a cDNA library using the primers of MAML1-UTR-F and MAML1-UTR-R. The FL-MAML1 reporter was constructed by inserting the DNA fragment of *MAML1*-3′UTR into the pmirgol1 vector *via* Xba I/Not I sites (52, 58). All the cloned constructs were validated by DNA sequencing. The constructs applied for this study are listed in Table S1, and the primer sequences for PCR are listed in Table S2. The ssDNAs, siRNAs, and their nucleotide sequences are listed in Table S3. The ssDNAs including (AT_5_)_5_, (CTG)_15_, and the chimera (CTG)_15_+(AT_5_)_5_ were designed according to the base priority for Atx2 (85) and DDX6 (54) respectively.

All the primary antibodies used in this study and the secondary antibodies purchased from Jackson ImmunoResearch Laboratories are listed in Table S4.

### Cell culture, transfection, and Western blotting analysis

HEK 293T cells (RRID: CVCL_1926) and HeLa cells (RRID: CVCL_0030) were from the Cell Bank of the Chinese Academy of Sciences (Shanghai), and they were mycoplasma-free and authenticated using STR profiling. The cells were cultured in DMEM (Corning, Glendale) supplemented with 10% fetal bovine serum (BioInd, Kibbutz Beit-Haemek) and penicillin/streptomycin at 37 °C under a humidified atmosphere containing 5% (v/v) CO_2_.

Transfection of plasmids was performed by using PolyJet reagent (SignaGen) following the manufacturer’s instructions. Transfection of siRNA was carried out by using Lipofectamine 3000 (Invitrogen) diluted in serum-free DMEM. Then the diluted PolyJet or Lipofectamine 3000 reagent was mixed with the diluted plasmid solution. After incubated for 15 min at room temperature, the mixture was added to the medium and cultured for 6 h, then the original medium was replaced with fresh complete medium.

The protein samples or fractions from cell lysates were subjected to SDS-PAGE and then transferred onto PVDF membranes (Millipore). When needed, the blots were cut prior to antibody incubation. The indicated proteins were incubated with specific primary and secondary antibodies and detected by ECL detection kit (Thermo Fisher Scientific). All images were taken using Minichemi 610 Plus software (Sage Creation Science). For quantification, the integral grayscale values of protein bands were recorded by ImageJ software.

### Supernatant/pellet fractionation

About 48 h after transfection, the HEK 293T or HeLa cells were harvested and lysed with a RIPA buffer (50 mM Tris-HCl, pH 7.5, 150 mM NaCl, 1 mM EDTA, 1% NP-40, and cocktail protease inhibitor (Roche)) in 100 μl per sample on ice for 30 min, and then the lysates were centrifuged at 13,000 rpm for 15 min at 4 °C. The supernatant fraction was added with 4× loading buffer (8% SDS). The pellet fraction was washed with the RIPA buffer for three times and then added with 4× loading buffer (8% SDS). RNase A (Invitrogen) with a final concentration of 0.5 μg/μl or ssDNA (Sangon Biotech) with a final concentration of 5 μM was added before cell lysis. Equal volumes of the supernatant and pellet fractions were subjected to SDS-PAGE and Western blotting analysis.

### Immunofluorescence imaging

HEK 293T or HeLa cells were transfected with the indicated plasmids, about 48 h later the cells grown on glass coverslips were washed with a phosphate-buffered saline (PBS) buffer (10 mM Na_2_HPO_4_, 1.8 mM KH_2_PO_4_, 140 mM NaCl, 2.7 mM KCl, pH 7.3), and then fixed with 4% paraformaldehyde for 15 min, permeabilized with 0.1% Triton X-100, and blocked with the blocking solution (10% fetal bovine serum in PBS buffer) for 1 h at room temperature. The fixed cells were incubated with the respective primary antibodies at 4 °C overnight. After being washed with the PBS buffer, the cells were labeled with fluorescein isothiocyanate-conjugated (FITC) antibody or tetramethylrhodamine isothiocyanate-conjugated (TRITC) antibody (Jackson ImmunoResearch Laboratories), and the nuclei were stained with Hoechst 33342 (Thermo Fisher Scientific). The cells were visualized on an FV3000 confocal microscope (Olympus).

### Co-immunoprecipitation with RNase and/or ssDNA treatment

HEK 293T cells were harvested 48 h after transfection and lysed in a PEB buffer (10 mM HEPES, pH 7.4, 100 mM KCl, 5 mM MgCl_2_, 1 mM DTT, 0.1% NP-40, 1 mM PMSF, supplemented with cocktail protease inhibitor (Roche)) on ice for 30 min, and then centrifuged at 13,000 rpm for 20 min at 4 °C. The supernatant was added into the anti-HA beads (Abmart) that had been previously washed and then incubated for 2 h at 4 °C. The beads were washed with the PEB buffer three times (1400 rpm, 4 min) and then boiled in 50 μl of 2 × loading buffer (4% SDS). Then, the proteins were analyzed by immunoblotting. Similar experiments were also performed with RNase and/or ssDNA treatment, in which RNase A and/or ssDNA were added during the incubation process of the lysates and beads. RNase A was used with a final concentration of 0.5 μg/μl and ssDNA was used with that of 5 μM in this study.

### Lentiviral vector production and transduction

HEK 293T cells were seeded at a density of ∼80% confluency in a 10-cm dish. About 24 h later, the cells were transfected with 3 μg of PSAPX2.5, 3 μg of PDM 2.6 and 4 μg of pCDH-FLAG-Atx2_23Q_, pCDH-FLAG-Atx2_99Q_ or pCDH vector per dish diluted by Opti-MEM. After cultured for 6 h, the original medium was replaced by a fresh complete medium. Viral particles in the supernatant were harvested 48 h post-transfection and filtered through a 0.45-μm filter (Sarstedt), then the collected virus was stored at −80 °C. HEK 293 T cells were seeded at a density of 80 to 90% confluency in a 10-cm dish approximately 24 h before transfection. The thawed virus was diluted by fresh media in equal volumes in the presence of 4 mg/ml polybrene to transduce, and then 12 h later the medium was renewed. About 48 h after transfection, puromycin was added with a final concentration of 1 μg/μl to screen the transfected cells lasting for 48 h.

### Luciferase reporter assay

HEK 293T cells were seeded at a density of ∼70% confluency and transfected 24 h post-seeding with the indicated plasmid or siRNA, and about 24 h later the reporter plasmid was transfected. After cultured for another 24 h, the cells were harvested and lysed in the Lysis Buffer (YEASEN). The activities of the FL and RL luciferases were measured using Dual-Luciferase Reporter Assay (YEASEN), and the FL/RL ratio was applied to present the enzymatic activity. Simultaneously, the cell lysates were subjected to Western blotting to determine the relative protein expression levels. For the rescue assay, the indicated plasmids were firstly co-transfected into HEK 293T cells, and then 24 h later the reporter plasmid was transfected into the cells for another 24-h culture.

### Mis-splicing analysis

HEK 293T cells were seeded at a density of ∼70% confluency in a 6-well plate and transfected with a series of PQE Atx2-N317 variants 24 h post seeding, then the cells were harvested 48 h after transfection. One-half of the cell samples was applied to measure the percentage of mis-splicing of *Ppp2r5c* or *IR2*, while another half was subjected to Western blotting to examine the protein expression level. The total RNA was extracted with TRIzol reagent (Invitrogen), and the cDNAs were generated by retro-transcription kit using 4× Ezscript Reverse Transcription Mix Ⅱ (EZBioscience). The PCR amplification was performed using the specific primers (Table S2), and *GAPDH* was used as an internal reference. Each PCR was performed in a reaction solution mixed with 0.2 μM of primers and the standard Taq-polymerase (TIANGEN) for 28 cycles (annealing temperature at 58 °C). Mouse monoclonal anti-FLAG (Sigma) and anti-GAPDH (Proteintech) antibodies were used for Western blotting analysis. The images were taken using Scion Image software (Scion Corp). For quantification, the integral grayscale values of the protein bands were recorded by ImageJ software.

### RT-qPCR assay

HEK 293T cells were seeded at a density of ∼70% confluency in a 6-well plate and harvested 48 h after transfection. The total RNA was extracted with TRIzol reagent (Invitrogen), and the cDNAs were generated by retro-transcription kit using 4 × Ezscript Reverse Transcription Mix Ⅱ (EZBioscience). The PCR amplification was performed by using the specific primers (Table S2), and *GAPDH* was used as an internal reference. The data were recorded with two technical replicates for each sample with the mean Ct values being analyzed for three biological replicates by Roche LightCycler96 PCR (Roche).

### Quantification and statistical analysis

For quantification of Western blots, the integral grayscale values of indicated protein bands were obtained using ImageJ software and normalized to that of the respective control. For quantification of the number of P-bodies, the confocal images were imported into ImageJ software, and the number of dots was recorded using its particle analysis module. For co-localization analysis of the indicated proteins, the fluorescence intensity was recorded using ImageJ and plotted by GraphPad Prism 8.0 software.

The data were obtained from at least three independent experiments and then presented as Mean ± SD. Statistical analysis was performed in GraphPad Prism 8.0 using Student’s *t* test. Differences were considered statistically significant at *p* < 0.05. In all experiments, the *p*-values were labeled in the graphs with ∗ (*p* < 0.05), ∗∗ (*p* < 0.01), ∗∗∗ (*p* < 0.001), or N.S. (no significance).

## Data availability

The data supporting the findings of this study are available from the corresponding author and the source data for the main figures and supplementary figures could be provided upon reasonable request.

## Supporting information

This article contains supporting information.

## Conflict of interest

The authors declare that they have no known competing financial interests or personal relationships that could have appeared to influence the work reported in this paper.
