# Supplementary material for: PolyQ-expanded ataxin-2 aggregation impairs cellular processing-body homeostasis via sequestering the RNA helicase DDX6
Source: J Biol Chem. 2024 May 27;300(7):107413. doi: 10.1016/j.jbc.2024.107413 (PMC11254730; doi:10.1016/j.jbc.2024.107413)
Supplement: Supporting Tables Figures [file mmc1.pdf]

Supporting Information Wang, et al.

PolyQ-expanded ataxin-2 aggregation impairs cellular processing-body homeostasis via sequestering the RNA helicase DDX6

Jian-Yang Wang[1,2], Ya-Jun Liu[1,2], Xiang-Le Zhang[1,2], Yin-Hu Liu[1,2], Lei-Lei Jiang[1], Hong-Yu Hu[1,*]

1 State Key Laboratory of Molecular Biology, Shanghai Institute of Biochemistry and Cell Biology, Center for Excellence in Molecular Cell Science, Chinese Academy of Sciences, Shanghai 200031, P. R. China.

2 University of Chinese Academy of Sciences, Beijing 100049, P. R. China.

* For correspondence: Hong-Yu Hu, hyhu@sibcb.ac.cn.

Running Title： Ataxin-2 aggregation on P-body homeostasis

Figure S1

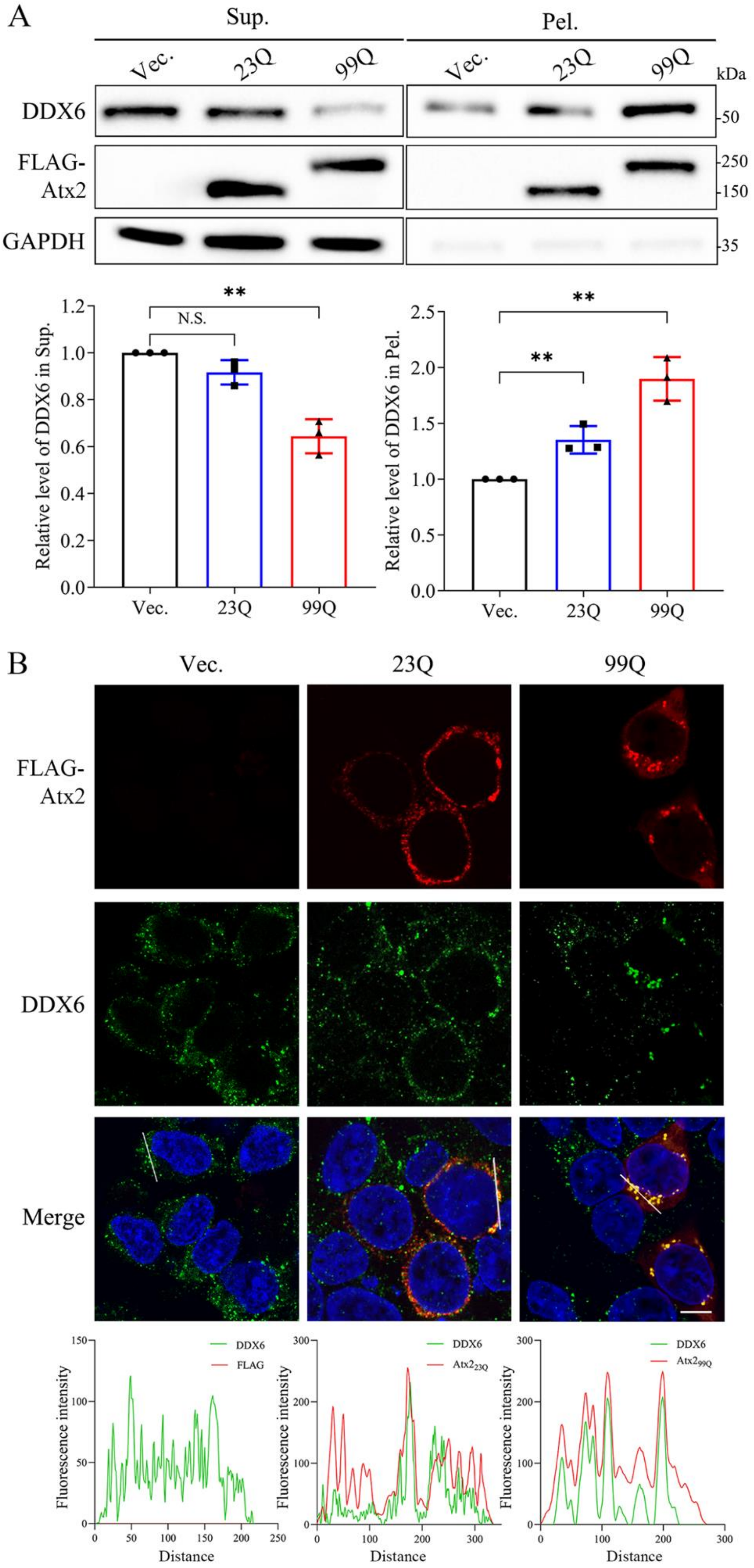
A
Sup.
Pel.
Vec.
23Q
99Q
kDa
DDX6
FLAG-
Atx2
GAPDH
50
250
150
35
Relative level of DDX6 in Sup.
Relative level of DDX6 in Pel.
N.S.
**
B
FLAG-
Atx2
DDX6
Merge
DDX6
FLAG
Atx2 23Q
Atx2 99Q
Fluorescence intensity
Distance

Figure S1. Sequestration of endogenous DDX6 by PQE Atx2 in HEK 293T cells. (A) S/P fractionation for characterizing sequestration of endogenous DDX6 into Atx2 aggregates. HEK 293T cells were transfected with each indicated plasmid, and after cultured for 48 hrs the cells were collected and lysed. The lysates were then subjected to S/P fractionation with Western blotting analysis. Vec., vector; 23Q, $Atx2_{23Q}$; 99Q, $Atx2_{99Q}$; Sup., supernatant; Pel., pellet. Data are shown as Mean ± SD (n=3). **, $p<0.01$; N.S., no significance. (B) Immunofluorescence imaging for co-localization of DDX6 with Atx2. HEK 293T cells were transfected with each indicated plasmid, and after cultured for 48 hrs the cells were fixed and immunostained with indicated antibodies. Atx2 was stained with anti-FLAG antibody (red), DDX6 was stained with anti-DDX6 antibody (green), and nuclei were stained with Hoechst (blue). Scale bar = 10 µm.

Figure S2

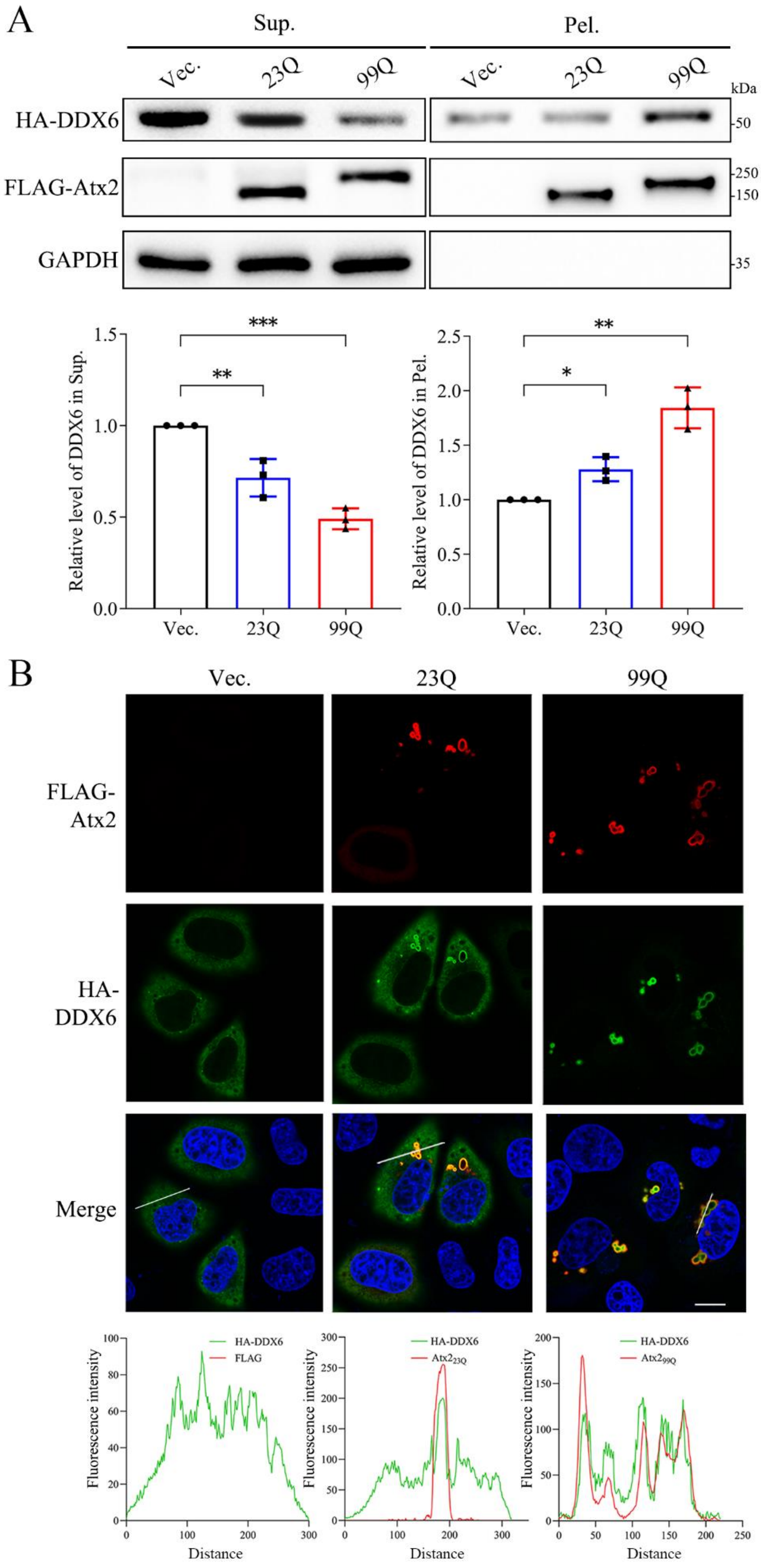

A
Sup.
Pel.
Vec.
23Q
99Q
kDa
HA-DDX6
FLAG-Atx2
GAPDH
50
250
150
35
Relative level of DDX6 in Sup.
Relative level of DDX6 in Pel.
***
**
*
B
Vec.
23Q
99Q
FLAG-Atx2
HA-DDX6
Merge
HA-DDX6
FLAG
Atx2 23Q
Atx2 99Q
Fluorescence intensity
Distance

Figure S2. Sequestration of exogenous DDX6 by PQE Atx2. (A) S/P fractionation for characterizing sequestration of exogenous DDX6 by PQE Atx2. HeLa cells were co-transfected with each indicated plasmid and collected after 48-hr culture, then the cell lysates were subjected to S/P fractionation with Western blotting analysis for HA-DDX6. Data are shown as Mean ± SD (n=3). *, $p<0.05$; **, $p<0.01$; ***, $p<0.001$. (B) Immunofluorescence imaging for co-localization of exogenous DDX6 with PQE Atx2. HeLa cells were co-transfected with each indicated plasmid and collected after 48-hr culture. Atx2 was stained with anti-FLAG antibody (red), HA-DDX6 was stained with anti-HA antibody (green), and nuclei were stained with Hoechst (blue). Scale bar = 10 μm.

Figure S3

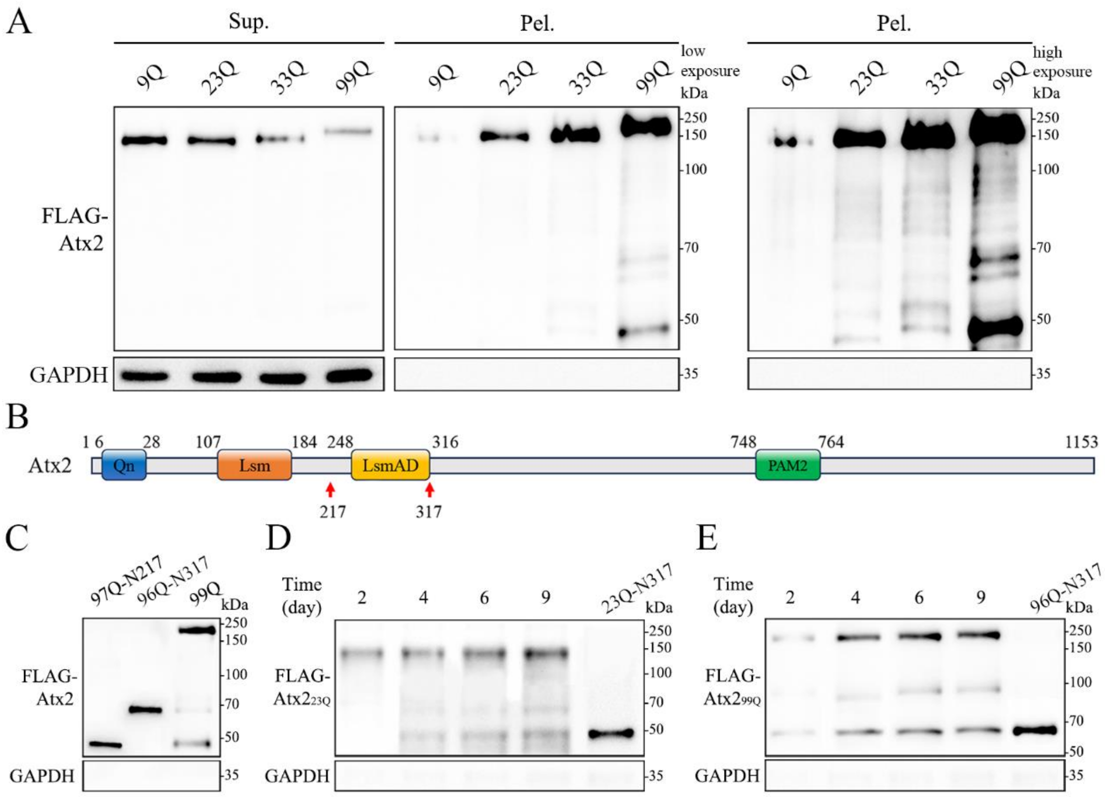

Figure S3. Degradation of PQE Atx2 into N-terminal fragments. (A) S/P fractionation for characterizing aggregation of PQE Atx2. HEK 293T cells were transfected with each indicated plasmid and collected after 48-hr culture, then the cell lysates were subjected to S/P fractionation with Western blotting analysis. There were two proteolytic bands with the estimated M.W. of ~65-kDa and ~45-kDa respectively in the pellet fraction as shown in the gel. (B) Domain architecture and the possible cleavage sites in the full-length sequence of Atx2. (C) S/P fractionation for characterizing the proteolytic fragments of FLAG-Atx2$_{99Q}$ in the pellet. The N-terminal truncations were set as markers. 96Q-N317, Atx2$_{96Q}$-N317; 97Q-N217, Atx2$_{97Q}$-N217. (D, E) S/P fractionation for characterizing degradation of the stably expressed Atx2. The expression plasmid was constructed by using the lentiviral vector pCDH-CMV-MCS-EF1-Puro and transfected into HEK 293T cells. The cells were collected at different culture times and the lysates were subjected to S/P fractionation with Western blotting for analyzing the degradation of FLAG-Atx2$_{23Q}$ (D) or FLAG-Atx2$_{99Q}$ (E). The N-terminal fragments in the pellet fraction were shown in the gels.

Figure S4

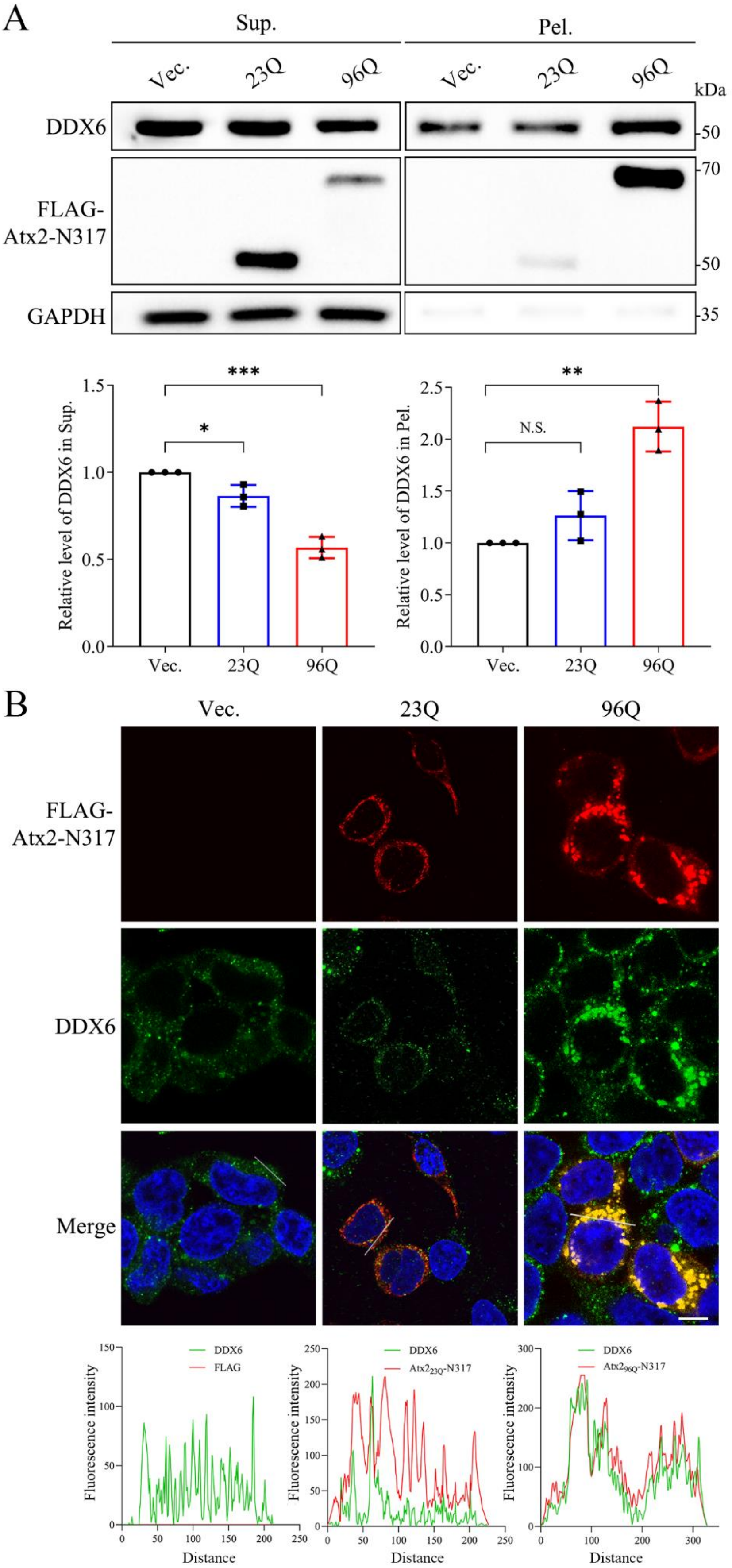

A
Sup.
Pel.
Vec.
23Q
96Q
kDa
DDX6
FLAG-
Atx2-N317
GAPDH
50
70
50
35
***
*
**
N.S.
Relative level of DDX6 in Sup.
Relative level of DDX6 in Pel.
B
Vec.
23Q
96Q
FLAG-
Atx2-N317
DDX6
Merge
DDX6
FLAG
Atx2$_{23Q}$-N317
Atx2$_{96Q}$-N317
Fluorescence intensity
Distance

Figure S4. Sequestration of endogenous DDX6 by PQE Atx2-N317 in HEK 293T cells. (A) S/P fractionation for characterizing sequestration of endogenous DDX6 into Atx2-N317 aggregates. HEK 293T cells were transfected with each indicated plasmid and further cultured for 48 hrs. The cell lysates were subjected to S/P fractionation with Western blotting analysis. Vec., vector; 23Q, $Atx2_{23Q}$-N317; 96Q, $Atx2_{96Q}$-N317. Data are shown as Mean ± SD (n=3). *, $p<0.05$; **, $p<0.01$; ***, $p<0.001$; N.S., no significance. (B) Immunofluorescence imaging for co-localization of DDX6 with Atx2-N317. HEK 293T cells were transfected with each indicated plasmid, and after cultured for 48 hrs the cells were fixed and immunostained with indicated antibodies. Atx2-N317 was stained with anti-FLAG antibody (red), DDX6 was stained with anti-DDX6 antibody (green), and nuclei were stained with Hoechst (blue). Scale bar = 10 μm.

Figure S5

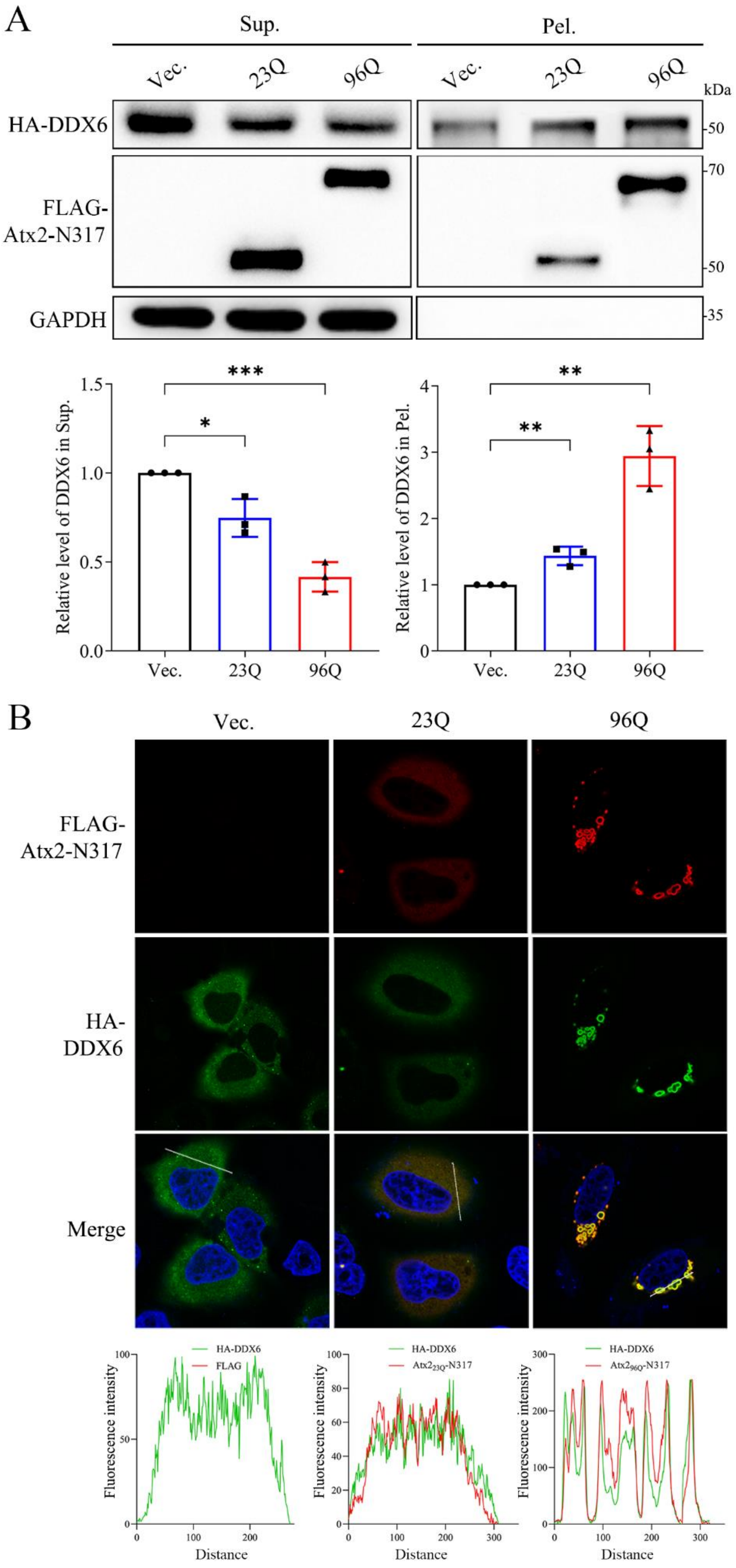
A
Sup.
Pel.
Vec.
23Q
96Q
kDa
HA-DDX6
FLAG-
Atx2-N317
GAPDH
50
70
50
35
Relative level of DDX6 in Sup.
Relative level of DDX6 in Pel.
***
*
**
**
B
Vec.
23Q
96Q
FLAG-
Atx2-N317
HA-
DDX6
Merge
HA-DDX6
FLAG
Atx2$_{23Q}$-N317
Atx2$_{96Q}$-N317
Fluorescence intensity
Distance

Figure S5. Sequestration of exogenous DDX6 by PQE Atx2-N317. (A) S/P fractionation for characterizing sequestration of exogenous DDX6 by PQE Atx2-N317. HeLa cells were co-transfected with each indicated plasmid and collected after 48-hr culture. The cell lysates were subjected to S/P fractionation with Western blotting analysis for HA-DDX6. Data are shown as Mean ± SD (n=3). *, $p<0.05$; **, $p<0.01$; ***, $p<0.001$. (B) Immunofluorescence imaging for co-localization of exogenous DDX6 with PQE Atx2-N317. HeLa cells were co-transfected with each indicated plasmid and collected after 48-hr culture. Atx2 was stained with anti-FLAG antibody (red), HA-DDX6 was stained with anti-HA antibody (green), and nuclei were stained with Hoechst (blue). Scale bar = 10 μm.

Figure S6

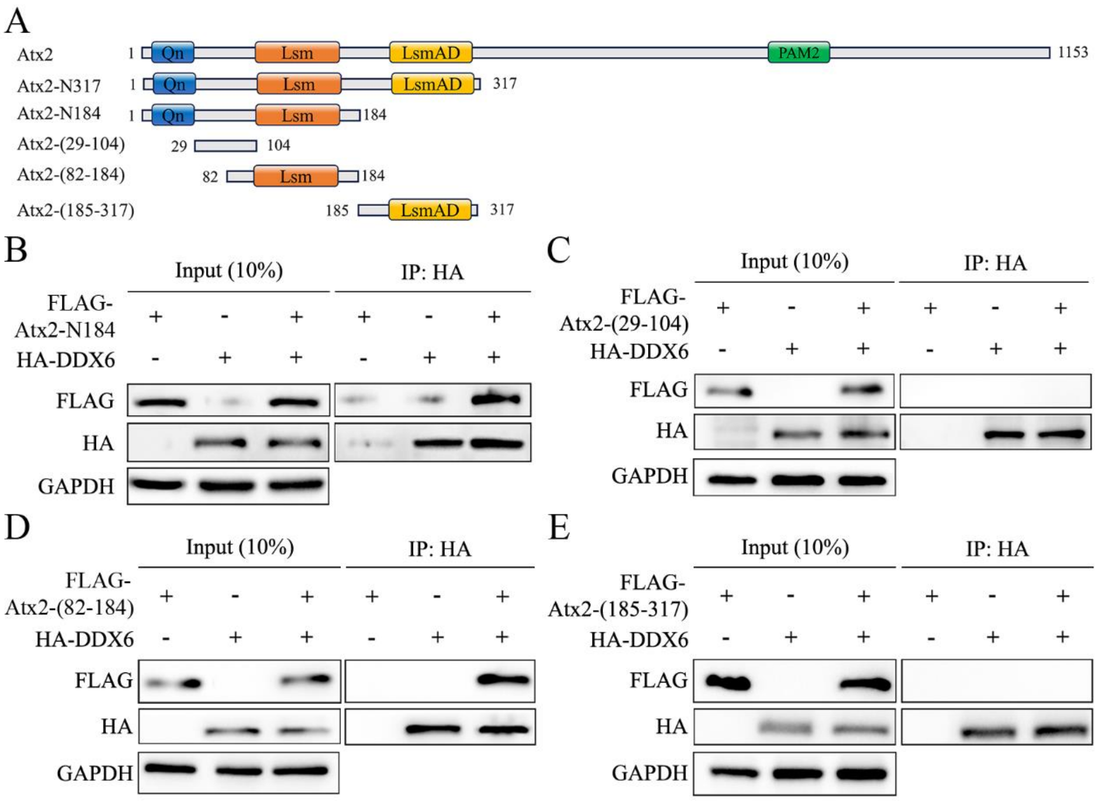

Figure S6. Characterization of the domain in Atx2 responsible for interacting with DDX6. (A) Schematic diagrams of full-length Atx2 and its indicated truncations. (B, C, D, E) Characterization of various Atx2 fragments interacting with DDX6 by co-IP assay. HEK 293T cells were co-transfected with FLAG-tagged Atx2-N184 (B), Atx2-(29-104) (C), Atx2-(82-184) (D) or Atx2-(185-317) (E), and HA-DDX6. After cultured for 48 hrs, the cell lysates were subjected to co-IP assay with anti-HA agarose beads. Atx2-N184, N-terminal residues 1 - 184; Atx2-(29-104), residues 29 - 104; Atx2-(82-184), residues 82 - 184; Atx2-(185-317), residues 185 - 317.

Figure S7

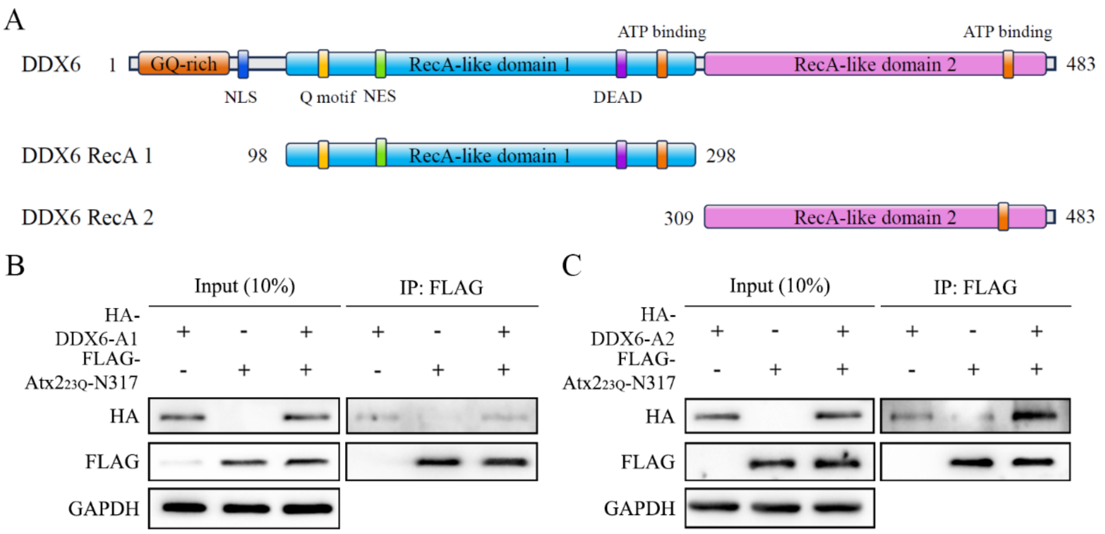

Figure S7. Characterization of the domain in DDX6 responsible for interacting with Atx2. (A) Schematic diagrams of DDX6 and its indicated fragments. (B, C) Characterization of the DDX6 fragments interacting with Atx2-N317 by co-IP assay. HEK 293T cells were co-transfected with HA-tagged DDX6-A1 (B) or DDX6-A2 (C) and FLAG-Atx2$_{23Q}$-N317. After culture for 48 hrs, the cell lysates were subjected to co-IP assay with anti-FLAG agarose beads. DDX6-A1, the RecA1 portion of DDX6, residues 98 - 298; DDX6-A2, the RecA2 portion of DDX6, residues 309 - 483.

Figure S8

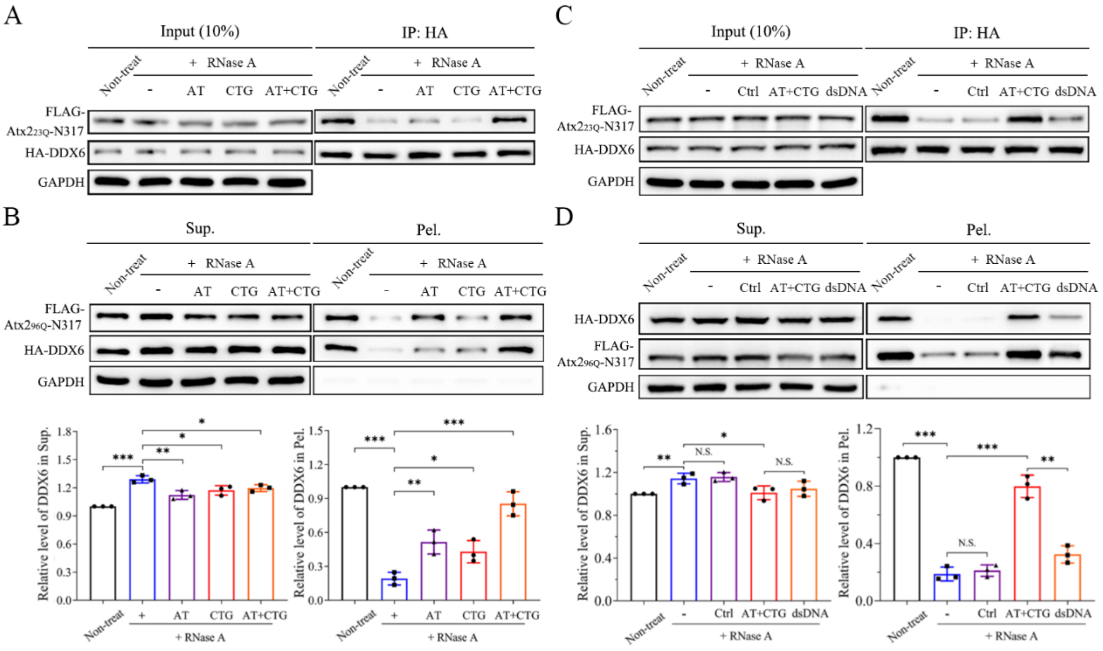

Figure S8. Examination of the role of RNA in mediating Atx2-DDX6 interaction and sequestration. (A, C) Co-IP experiment examining the effects of RNase and ssDNA or dsDNA treatments on the interaction between FLAG-Atx2$_{23Q}$-N317 and HA-DDX6. (B, D) S/P fractionation for characterizing the effects of RNase and ssDNA or dsRNA treatments on the sequestration of HA-DDX6 by FLAG-Atx2$_{96Q}$-N317. The treatments include non-treat, RNase-A treatment, and RNase-A plus ssDNA or dsDNA treatments. AT, (AT$_5$)$_5$; CTG, (CTG)$_{15}$; AT+CTG, (AT$_5$)$_5$+(CTG)$_{15}$; Ctrl, control ssDNA with a scrambled sequence; dsDNA, double-stranded DNA from (AT)$_5$+(CTG)$_{15}$. HEK 293T cells were transfected with each indicated plasmid, after 48 hrs the cell lysates were treated with RNase A and ssDNA or dsDNA, and then subjected to Co-IP (A, C) or S/P fractionation (B, D) experiment with Western blotting for DDX6. Data are shown as Mean ± SD (n=3). *, $p<0.05$; **, $p<0.01$; ***, $p<0.001$; N.S., no significance.

Figure S9

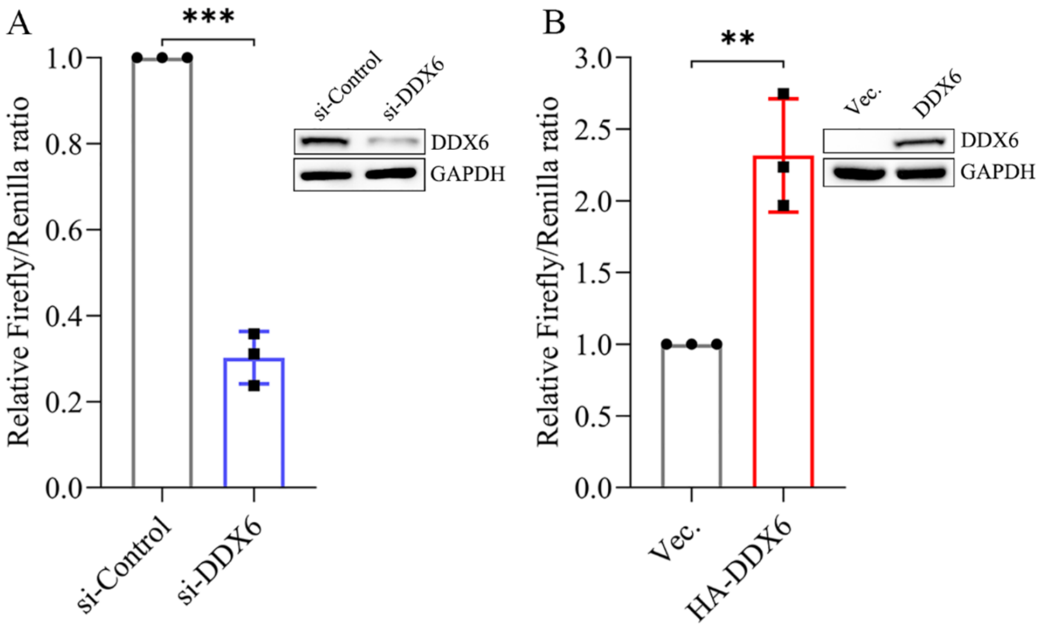

Figure S9. Validation of the FL-MAML1 reporter. (A) Relative FL/RL ratio of the FL-MAML1 reporter upon knockdown of DDX6. HEK 293T cells were transfected with each indicated plasmid and collected after 48-hr culture, then the cell lysates were subjected to detection of the luciferase activities. The inset showed the knockdown of DDX6 by siRNA interference. Data are shown as Mean ± SD (n=3). ***, $p<0.001$. (B) Relative FL/RL ratio of the FL-MAML1 reporter upon overexpression of DDX6. The inset showed the overexpression of exogenous HA-DDX6. Data are shown as Mean ± SD (n=3). **, $p<0.01$.

Figure S10

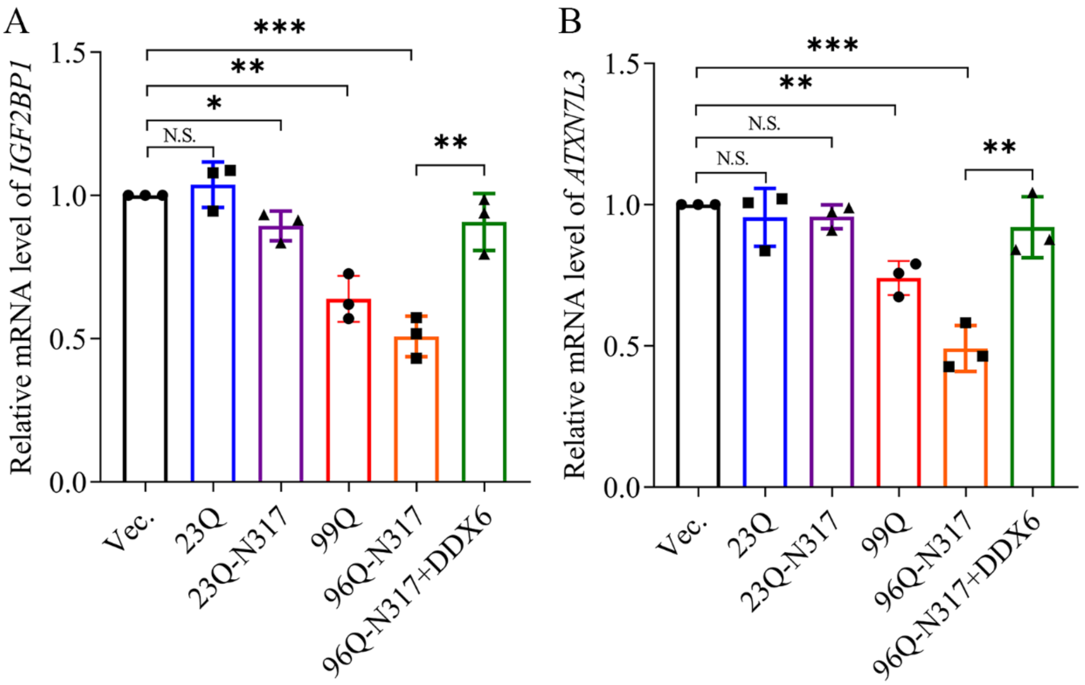

Figure S10. PQE Atx2 and Atx2-N317 reduce the target mRNA levels through endonuclease MARF1. (A, B) Endogenous mRNA level of *IGF2BP1* (A) or *ATXN7L3* (B) in the cells expressing PQE Atx2 or Atx2-N317. HEK 293T cells were transfected with each indicated plasmid and collected after 48-hr culture. The extracted RNA was reversely transcribed into cDNA and detected by quantitative PCR for *IGF2BP1* and *ATXN7L*, respectively. The mRNA levels were normalized to *GAPDH* and set the vector as a control. Data are shown as Mean ± SD (n=3). *, p<0.05; **, p<0.01; ***, p<0.001; N.S., no significance.

Table S1. List of the constructs applied in this study.

Constructs	Vectors	Restriction enzyme sites	Additional
FLAG-Atx2$_{23Q}$	FLAG-pcDNA3.1	BamH I/Not I	23 Gln, FLAG tag
FLAG-Atx2$_{9Q}$	FLAG-pcDNA3.1	BamH I/Not I	9 Gln
FLAG-Atx2$_{33Q}$	FLAG-pcDNA3.1	BamH I/Not I	33 Gln
FLAG-Atx2$_{99Q}$	FLAG-pcDNA3.1	BamH I/Not I	99 Gln
FLAG-Atx2$_{3Q}$	FLAG-pcDNA3.1	Not I/Xba I	3 Gln,
FLAG-Atx2$_{23Q}$-N317	FLAG-pcDNA3.1	BamH I/Not I	Residues 1-317, 23 Gln
FLAG-Atx2$_{9Q}$-N317	FLAG-pcDNA3.1	BamH I/Not I	Residues 1-317, 9 Gln
FLAG-Atx2$_{33Q}$-N317	FLAG-pcDNA3.1	BamH I/Not I	Residues 1-317, 33 Gln
FLAG-Atx2$_{96Q}$-N317	FLAG-pcDNA3.1	BamH I/Not I	Residues 1-317, 96 Gln
FLAG-Atx2$_{97Q}$-N217	FLAG-pcDNA3.1	BamH I/Not I	Residues 1-217, 97 Gln
FLAG-Atx2-N184	FLAG-pcDNA3.1	BamH I/Not I	N-terminal residues 1-184
FLAG-Atx2-(29-104)	FLAG-pcDNA3.1	BamH I/Not I	Residues 29-104
FLAG-Atx2-(82-184)	FLAG-pcDNA3.1	BamH I/Not I	Residues 82-184
FLAG-Atx2-(185-317)	FLAG-pcDNA3.1	BamH I/Not I	Residues 185-317
HA-DDX6	HA-pcDNA3.1	BamH I/Not I	HA tag
HA-DDX6-A1	HA-pcDNA3.1	BamH I/Not I	HA tag
HA-DDX6-A2	HA-pcDNA3.1	BamH I/Not I	HA tag
HA-LSM14A	HA-pcDNA3.1	BamH I/Not I	HA tag
HA-4E-T	HA-pcDNA3.1	BamH I/Not I	HA tag
FL-MAML1	pmirgol1	Xba I/Not I	Dual-fluorescence reporter
FLAG-Htt$_{100Q}$-N552	FLAG-pcDNA3.1	BamH I/Xho I	FLAG tag
pCDH-Atx2$_{23Q}$	pCDH-puro	Xba I/Not I	23 Gln, FLAG tag
pCDH-Atx2$_{99Q}$	pCDH-puro	Xba I /Not I	99 Gln, FLAG tag

Table S2. Nucleotide sequences of the PCR primers applied in this study.

PCR primer	Sequence	Note
Atx2$_{3Q}$-F	ATAAGAATGCGGCCGCATGCAGCAGCAGCCTCCTCCT GCT	Not I site
Atx2$_{3Q}$-R	GCTCTAGATTACAACTGCTGTTGGTGGT	Xba I site
Atx2-F	GACGATAAAATCGATGGATCCATGAGC	BamH I site
Atx2-R	TAGATGCATGCTCGAGCGGCCGCTTACAACTGCTGTT GGTGGTGGG	Not I site
Atx2-N317-R	TAGATGCATGCTCGAGCGGCCGCTTAATTTCTCTGAAC TGCTGTGTATTTTTCTTC	Not I site
Atx2-N217-R	TAGATGCATGCTCGAGCGGCCGCTTAACCTGCATCCC AGGGCTCCA	Not I site
Atx2-N184-R	TAGATGCATGCTCGAGCGGCCGCTTACATATCTTTAAA CTGTACCACAACAAAGTC	Not I site
Atx2-29-F	CGGGATCCATGCCTCCTCCTGCTGCTGCTAAT	BamH I site
Atx2-82-F	CGGGATCCATGCTGGGCAGAGGTCGAAACAGT	BamH I site
Atx2-N185-F	GACGATAAAATCGATGGATCCGACTCCAGTTATGCAA AAAGAGATGCTT	BamH I site
Atx2-104-R	CGGAATTCTCATGCATAGATTCCATCAAAAGAAAT	EcoR I site
Atx2-184-R	CGGAATTCTCAGTCCATATCTTTAAACTGTACCA	EcoR I site
DDX6-F	GACGATAAAATCGATGGATCCATGAGCACGGCCAGAA CAGA	BamH I site
DDX6-R	TAGATGCATGCTCGAGCGGCCGCTTAAGGTTTCTCATC TTCTACAGGCTCG	Not I site
DDX6-A1-F	GACGATAAAATCGATGTTTGAAGATTACTGTTTGAAAC GGGAGT	BamH I site
DDX6-A1-R	TAGATGCATGCTCGAGCGGCCGCTTAAATCTCATAGGG TTTCTGCAAATGG	Not I site
DDX6-A2-F	GACGATAAAATCGATGGTAACCCAGTACTACGCATATG TAACTG	BamH I site
LSM14A-F	GTCCCCGACTACGCCGGATCCATGAGCGGGGGCACCC CT	BamH I site
LSM14A-R	TAGATGCATGCTCGAGCGGCCGCTCAGAGACTTGTTT GTAGACTATGCAGCAA	Not I site
4E-T-F	GTCCCCGACTACGCCGGATCCATGGATAGGAGAAGTA TGGGTGAAAC	BamH I site

4E-T-R	TAGATGCATGCTCGAGCGGCCGCTCACTGTCGGTATTCCAATTCATCTA	Not I site
MAML1-UTR-F	CGGCGCCGCTCTAGAGGTGTTGGGACAGCAGGATA	Xba I site
MAML1-UTR-R	CGGCGCCGCGCGGCCGCCATAGCTCCCCCAAAACACAC	Not I site
pCDH-Atx2-F	ACCTCCATAGAAGATTCTAGAGGTACCGAGCTCGGATCGC	Xba I site
pCDH-Atx2-R	GATCGCAGATCCTTCGCGGCCGCTTACAACTGC	Not I site
qPCR primer		
MAML1-F	CCAGTACCAAGACCCGACAC	
MAML1-R	GTTTTGGGAGCCAGGGAACT	
NOTCH2-F	GCACTCAGGTGTCTGCATCA	
NOTCH2-R	ATCCATGCACTGACCACCATT	
IGF2BP1-F	GCGATGAAGGCCATCGAAAC	
IGF2BP1-R	AGCTTCATGATGGCTTGCCT	
ATXN7L3-F	TCTGCATCAAACACCCTTCCC	
ATXN7L3-R	GGAGCCTGAATCAGAGGGTG	
Ppp2r5c-F	GGGAAGAAGCATGGGTTAAA	
Ppp2r5c-R	CTTCCAAGGCTTTCTTGGTG	
IR2-F	CCAAAGACAGACTCTCAGAT	
IR2-R	AACATCGCCAAGGGACCTGC	

Table S3. Nucleotide sequences of ssDNAs and siRNAs applied in this study.

Nucleotide	Sequence	Note
ssDNA		
$(AT_5)_5$	ATTTTTATTTTTATTTTTATTTTTATTTTT	ATTTTT repeat
$(CTG)_{15}$	CTGCTGCTGCTGCTGCTGCTGCTGCTGCTGC TGCTGCTGCTGCTG	CTG repeat
$(AT_5)_5$+$(CTG)_{15}$	ATTTTTATTTTTATTTTTATTTTTATTTTTCTGC TGCTGCTGCTGCTGCTGCTGCTGCTGCTGCT GCTGCTGCTG	ATTTTT and CTG repeats
N_{75}	(ATCG) scrambled sequence	Control ssDNA
dsDNA	5' ATTTTTATTTTTATTTTTATTTTTATTTTT<br>3' TAAAAATAAAAATAAAAATAAAAATAAAAA<br>CTGCTGCTGCTGCTGCTGCTGCTGCTGCTG<br>GACGACGACGACGACGACGACGACGACGAC<br>CTGCTGCTGCTGCTG 3'<br>GACGACGACGACGAC 5'	dsDNA from $(AT_5)_5$+$(CTG)_{15}$
siRNA		
si-Control	UUCUCCGAACGUGUCACGUTT	
siRNA-DDX6	GAGUUACUGAUGGGAAUUU	

Table S4. List of the antibodies used in this study.

Antibody	Source	Catalog No.
Anti-FLAG (mouse)	Sigma-Aldrich	F1804
Anti-HA (mouse)	Sigma-Aldrich	H3663
Anti-DDX6 (rabbit)	Novus Biologicals	NB200-191,
Anti-EDC4 (rabbit)	Novus Biologicals	NBP2-13944
Anti- MARF1(mouse)	Proteintech	55493-1-AP
Anti-NOTCH2 (rabbit)	Proteintech	28580-1-AP
Anti-GAPDH (mouse)	Proteintech	60004-1-Ig
Peroxidase AffiniPure Goat Anti-Mouse IgG (H+L)	Jackson ImmunoResearch Laboratories	115-035-003
Peroxidase AffiniPure Goat Anti-Rabbit IgG (H+L)	Jackson ImmunoResearch Laboratories	111-001-003
FITC IgG Fluorescein-Conjugated AffiniPure Goat Anti-Rabbit IgG(H+L)	ZsBIO	ZF-0311
TRITC IgG Fluorescein-Conjugated AffiniPure Goat Anti-Rabbit IgG(H+L)	ZsBIO	ZF-0316
FITC IgG Fluorescein-Conjugated AffiniPure Goat Anti-Mouse IgG(H+L)	ZsBIO	ZF-0312
TRITC IgG Fluorescein-Conjugated AffiniPure Goat Anti-Mouse IgG(H+L)	ZsBIO	ZF-0313
